# *Cinnamomi ramulus* inhibits cancer cells growth by inducing G2/M arrest

**DOI:** 10.3389/fphar.2023.1121799

**Published:** 2023-03-17

**Authors:** Jing Li, Hsi-Yuan Huang, Yang-Chi-Dung Lin, Huali Zuo, Yun Tang, Hsien-Da Huang

**Affiliations:** ^1^ School of Medicine, The Chinese University of Hong Kong, Shenzhen, Guangdong, China; ^2^ Warshel Institute for Computational Biology, The Chinese University of Hong Kong, Shenzhen, Guangdong, China

**Keywords:** *Cinnamomi ramulus*, transcriptomic analysis, differential expression analysis (DE), gene set enrichment analysis (GSEA), cell cycle

## Abstract

**Introduction:** *Cinnamomi ramulus* (CR) is one of the most widely used traditional Chinese medicine (TCM) with anti-cancer effects. Analyzing transcriptomic responses of different human cell lines to TCM treatment is a promising approach to understand the unbiased mechanism of TCM.

**Methods:** This study treated ten cancer cell lines with different CR concentrations, followed by mRNA sequencing. Differential expression (DE) analysis and gene set enrichment analysis (GSEA) were utilized to analyze transcriptomic data. Finally, the *in silico* screening results were verified by *in vitro* experiments.

**Results:** Both DE and GSEA analysis suggested the Cell cycle pathway was the most perturbated pathway by CR across these cell lines. By analyzing the clinical significance and prognosis of G2/M related genes (PLK1, CDK1, CCNB1, and CCNB2) in various cancer tissues, we found that they were up-regulated in most cancer types, and their down-regulation showed better overall survival rates in cancer patients. Finally, *in vitro* experiments validation on A549, Hep G2, and HeLa cells suggested that CR can inhibit cell growth by suppressing the PLK1/CDK1/ Cyclin B axis.

**Discussion:** This is the first study to apply transcriptomic analysis to investigate the cancer cell growth inhibition of CR on various human cancer cell lines. The core effect of CR on ten cancer cell lines is to induce G2/M arrest by inhibiting the PLK1/CDK1/Cyclin B axis.

## 1 Introduction

*Cinnamomi ramulus* (CR) or “Guizhi” is dried twigs of *Cinnamomum cassia* (L.) Presl, which has been used in clinics frequently for thousands of years, with the property of relieving exterior syndrome by diaphoresis, warming and smoothing the meridian, reinforcing Yang to transform Qi (Rather et al., 2016). CR was first recorded in the Traditional Chinese Medicine (TCM) book “Treatise on Cold Pathogenic and Miscellaneous Diseases” in Han Dynasty, and it has been recorded in the Chinese pharmacopeia since 1973. In TCM theory, CR is sweet in taste and warm in nature. According to statistics, around 65 Traditional Chinese Medicine formulas containing CR have been collected in the Chinese pharmacopeia, including Gui Zhi Fu Ling Wan, Waigan Fenghan Keli, Wu Mei Wan, etc. Up to now, over 100 chemical compounds have been identified from CR. Modern pharmacological studies have proved that CR has antibacterial, anti-inflammatory, antipyretic, and antiviral effects (Liu et al., 2020a). Clinically, CR is rarely used in a single prescription and is often used in combination with other herbs to treat diseases. Recently, several studies revealed that the extracts/monomers from CR possess anti-tumor effects against various cancer cells. Park et al. (2018) reported that the aqueous extracts of CR could inhibit human colorectal cancer cell viability by down-regulating cyclin D1 *via* GSK3β-dependent T286 phosphorylation and downregulation of β-catenin. Moreover, CR could also drive ROS-dependent apoptosis in human colorectal cancer cells. Pan et al. (2022) reported oral administration of CR inhibited the growth of colon cancer cells in C57BL/6 mice *via* Akt/ERK signaling pathways. In summary, CR is a widely used medicine in TCMs, but the underlying molecular mechanism inhibiting cancer cell growth remain largely unknown.

Transcriptomic data have extensively promoted the discovery of disease biomarkers and therapeutic drug targets (Yang et al., 2020), providing new clues for TCM research. Transcriptomic responses of human cancer cell lines to drug therapy is a promising approach to understanding complex responses to drug treatment (Iwata et al., 2019). Much research on TCM is currently based on traditional research methods, such as chemical analysis and complex pharmacological experiments, which is laborious and time-consuming. In recent years, researchers performed sequencing and bioinformatics analysis on herbal medicines with significant economic and medicinal value to clarify the mechanisms of action, extensively promoting the research and development of TCM (Chakraborty, 2018). Transcriptome analysis of mRNA reflects the collection of all mRNA products of a cell under a given condition (drug treatment or disease status), which provides a comprehensive view of biological changes resulting from multiple genetic variations. Therefore, altered transcriptome profiles can be used to elucidate the disease mechanisms and drug mode of action (Kwon et al., 2019). The application of transcriptomic data analysis combined with bioinformatics analysis in TCM research can significantly reduce the large number of experiments needed for screening pathways and therapeutic targets in the early stage of TCM research.

Cancer cell lines have been widely used as models for cancer studies. Many pharmaceutical companies have endorsed using suitable cell models to test anti-cancer drugs (Lopez-Camarillo, 2013). Typical perturbation experiments are always performed in specific cells to catch the responses by medicines that are often context-dependent. Hub transcriptomic signatures influenced by the drug are shared in multiple tissue types that remove specific backgrounds and can reflect more upstream pathway changes. Genome-wide expression profiles or perturbated expression profiles by drug treatment have been constructed and applied to drug discovery, including the Connectivity Map (cMap) and Cancer Cell Line Encyclopedia (CCLE). The cMap was based on 564 reference gene expression profiles generated by 164 compounds which are FDA-approved drugs treating human cell lines, including MCF7, PC3, SKMEL5, and HL60. A query gene signature in cMap is a short list of genes whose expression represents the biological state of interest. Then the query signature is compared to the reference expression dataset to find the similarity using the non-parametric, rank-based pattern-matching strategy (Lamb et al., 2006). Driven by TCGA, a database to characterize the genetic basis of human cancer, the CCLE performed sequencing on 947 human cancer cell lines and included 24 anti-cancer drug pharmacological profiles. CCLE is essential for anyone seeking to identify genetically related drug responses. It helps researchers capture clinically significant genomic features and use this information to accelerate clinical diagnosis and improve treatment. Even today, clinical trials are still costly, and there is an increasing need for reliable biomarkers and drug targets (Barretina et al., 2012).

**FIGURE 1:**
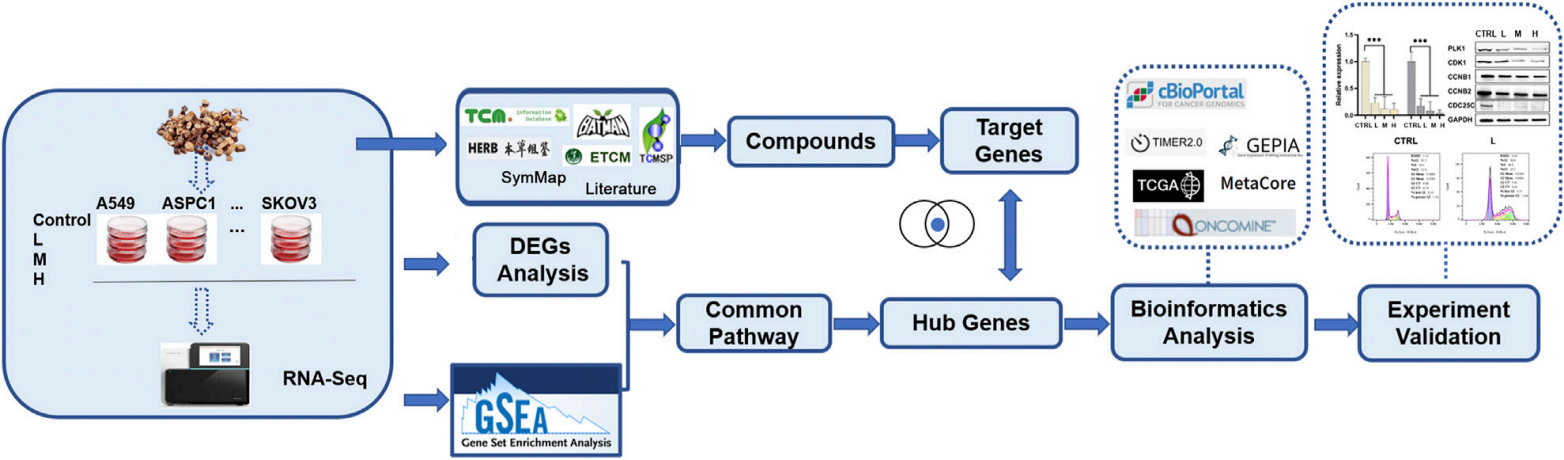
General workflow and research methods of this study.

In our group, we chose the ten widely studied cancer cell lines to test significant TCM drug effects across selected cancer cell lines, like cMap but can guide users to find the relationship between TCM drug, disease, and genes. And this study is a case study of the big project to explore the effects of *C. ramulus* on ten cancer cell lines. In this study, using RNA-seq data generated from ten cancer cells treated with CR and comprehensive transcriptomic analysis, we found cell cycle pathway was significantly associated with inhibition caused by CR. Then, we used a series of *in vitro* experiments to demonstrate that CR can dramatically inhibit the growth of ten cancer cell lines by blocking the PLK1/CDK1/Cyclin B axis. This is the first study applying transcriptomic analysis to explore the mechanism of CR on various human cancer cell lines. This study’s general workflow and research methods are shown in Figure 1; Supplementary Figure S1.

## 2 Materials and methods

### 2.1 UPLC-MS/MS analysis of CR granules

Analysis of CR chemical composition was performed using UPLC-MS/MS. UPLC conditions: Chromatographic separation was achieved using Vanquish Flex UHPLC (Thermo Fisher Scientific, Bremen, Germany) (Wang et al., 2021), and the chromatographic column used was ACQUITY UPLC T3 column (100 mm*2.1 mm, 1.7 µm, Waters, Milford, United States). The mobile phase consisted of solvent (A) 0.1% formic acid and (B) Acetonitrile. The gradient elution was as follows: 0–0.8 min, 2% B; 0.8–2.8 min, 2%–70% B; 2.8–5.6 min, 70%–90% B; 5.6–6.4 min, 90%–100% B; 6.4–8 min, 100% B; 8–8.1 min, 100%–2% B; 8.1–10 min, 2% B. MS conditions: Mass spectra were obtained on Q-Exactive (Thermo Scientific). Both positive and negative ion modes were performed on Q-Exactive (Wang et al., 2021).

### 2.2 Cell culture

Ten human cancer cell lines, including A549, HeLa, Hep G2, SKOV3, SKBR3, HCT116, MCF7, RBE, Hep 3B, and AsPC1, were obtained from CellCook Co. Ltd. (China, Guangzhou). Cells were incubated at 37°C and 5% CO_2_ in an incubator (Helgason and Miller, 2005).

### 2.3 CCK8 assay

CR granules were bought from E-FANG pharmaceutical (Guangdong, China) and dissolved in their corresponding culture medium. Then we performed CCK8 assay to detect the cell viability after CR treatment (Li et al., 2019). Cells were treated with a series of concentrations (3.3 × 10^−4^, 1 × 10^−3^, 3.3 × 10^−3^, 1 × 10^−2^, 3.3 × 10^−2^, 1 × 10^−1^, 3.3 × 10^−1^, and 1 g/mL) of CR for 24 h. After incubation, 110 μL fresh medium, including 10 μL CCK8 solution, was added to each well. The plates were incubated for an additional 2 hours at 37°C. Then the absorbance was measured at 450 nm wavelength by a microplate reader (BioTek).

### 2.4 CR gene targets identification

We collected the compound information from TCMSP (Ru et al., 2014) (https://old.tcmsp-e.com/tcmsp.php), SymMap (Wu et al., 2018) (http://www.symmap.org), ETCM(Xu et al., 2018) (http://www.tcmip.cn/ETCM/), TCM-ID (https://bidd.group/TCMID/), BATMAN (Liu et al., 2016) (http://bionet.ncpsb.org.cn/batman-tcm/), HERB (Fang et al., 2020) (http://herb.ac.cn/), and literature. Putative gene targets were collected from BioAssay Results from Pubchem. Moreover, we only collected active interactions between compounds and human genes. Finally, 857 compound-genes interactions from the 83 compounds and 341 distinct targets were obtained (Supplementary Table S1).

### 2.5 RNA extraction and next-generation sequencing

Total RNA was extracted from CR-treated cancer cell lines using Trizol reagent (Invitrogen) following the manufacturer’s protocol. We sequenced pair-end reads of 100 bp (PE100) on the BGISEQ-500 platform for subsequent data analysis (Zhu et al., 2018).

### 2.6 Transcriptome analysis and prognostic analysis

MRNA-seq raw data were processed by nf-core/rnaseq pipeline (v3.0) with the parameter of “–genome hg38 –gencode” to obtain the read count matrix for all samples. iDEP (http://ge-lab.org/idep/), integrated Differential Expression and Pathway analysis, was used to perform read counts normalization and differential expression analysis (Ge et al., 2018). Differential expression mRNA analysis was performed with DESeq2 and selected DEGs with FDR<0.05 and fold change≥2. Metascape (Zhou et al., 2019), Metacore TM (©Clarivate Analytics), and GSEA (Subramanian et al., 2005) were used for the functional enrichment analysis. For GSEA analysis, the c2.cp.kegg.v7.5. symbols.gmt (curated) gene sets database was used as the gene set collection for analysis. GSEA performed 1,000 permutations. The maximum and minimum sizes for gene sets were 500 and 15, respectively. The cutoff for significant gene sets was false discovery rate <25%. Nominal *p*-value < 0.05. Human Protein Atlas (Ponten et al., 2008) (HPA) was adopted to check the gene expression specificity in different cell lines and protein expression levels in tumor tissues. Timer (Li et al., 2017) and Oncomine (Rhodes et al., 2004) were used to check the expression level in clinical samples compared with non-cancer ones. Survival analyses were performed using the GEPIA (Tang et al., 2017). cBioportal (Gao et al., 2013) was used to analyze the hub genes’ genetic aberration in cancers.

### 2.7 Quantitative real-time PCR (qPCR) assay

QPCR was used to analyze the mRNA expression of CDK1, PLK1, CCNB1, CCNB2, and CDC25C in cancer cell samples. Total RNA extraction was performed with the Direct-zol RNA miniprep kit according to the manufacturer’s instructions. cDNA was generated using SuperScript III Reverse Transcriptase (Invitrogen). The total cDNA was mixed with SYBR Green and master mix, then loaded into QuantStudio™ 6 Flex Real-Time PCR System (Applied Biosystems) for amplification and detection (Polaski et al., 2021). The primer sequences are listed in Table 1.

**TABLE 1:** Primers used for qPCR assay.

Gene names	Sequences (5′-3′)
CDK1-F	GAT​GTG​CTT​ATG​CAG​GAT​TCC
CDK1-R	CAT​GTA​CTG​ACC​AGG​AGG​GAT​AG
PLK1-F	AGC​CCC​TCA​CAG​TCC​TCA​ATA​A
PLK1-R	TCG​ACC​ACC​TCA​CCT​GTC​TCT
CCNB1-F	AAG​TCA​TGG​AGA​ATC​TGC​TGC​AT
CCNB1-R	TGG​CAG​CAA​TCA​CAA​GAA​GAA
CCNB2-F	CTG​TAC​ATG​TGC​GTT​GGC​ATT
CCNB2-R	AAG​CCA​AGA​GCA​GAG​CAG​TAA​TC
CDC25C-F	GCG​GCT​ACA​GAG​ACT​TCT​TTC​C
CDC25C-R	CAC​CTC​AGC​AAC​TCA​GTC​TTG​TG
GAPDH-F	ACC​CAC​TCC​TCC​ACC​TTT​GAC
GAPDH-R	TGT​TGC​TGT​AGC​CAA​ATT​CGT​T

### 2.8 Western blotting

Western blot was performed as previously described (Kurien and Scofield, 2015). The equal amount of protein was separated with 10% SDS-PAGE and then transferred onto polyvinylidene difluoride membranes (Millipore, United States). Next, transferred membranes were blocked with 5% BSA in TBST buffer for 1 hour at room temperature. Then, membranes were probed with specific primary antibodies anti-CDK antibody (#ab18, ABCAM), anti-PLK1 antibody (#ab17056), anti-Cyclin B1 (#ab32053, ABCAM), anti-CyclinB2 (#ab185622, ABCAM), anti-CDC25C (#ab32444, ABCAM) and anti-GAPDH (#60004-1-lg, Proteintech) at 4°C overnight. Then, membranes were incubated with secondary antibodies conjugated to horseradish peroxidase (A0216/A0208, Beyotime, China). Finally, bands were visualized by enhanced chemiluminescence.

### 2.9 Cell cycle analysis

Cell cycle assay was performed using a Cell Cycles and Apoptosis Analysis Kit (C1052, Beyotime) (Luo et al., 2018). Cells were collected and fixed with 70% ice-cold ethanol at −20°C overnight. For cell cycle detection, cells were centrifuged, re-suspended in a mixture of 50 μL/mL propidium iodide (PI) and 20 μL/mL RNase A, and incubated at 37°C for 30 min. The red fluorescence was detected at the excitation wavelength of 488 nm by flow cytometry (CytoFlex).

### 2.10 Statistical analysis

All experiment data were presented as mean ± SD and analyzed using GraphPad Prism nine software. Statistical analysis of data was performed with Student’s t-test. Results with *p* < 0.05 were considered statistical significance.

## 3 Results

### 3.1 CR might be closely related to various cancers and cancer-related pathways

**FIGURE 2:**
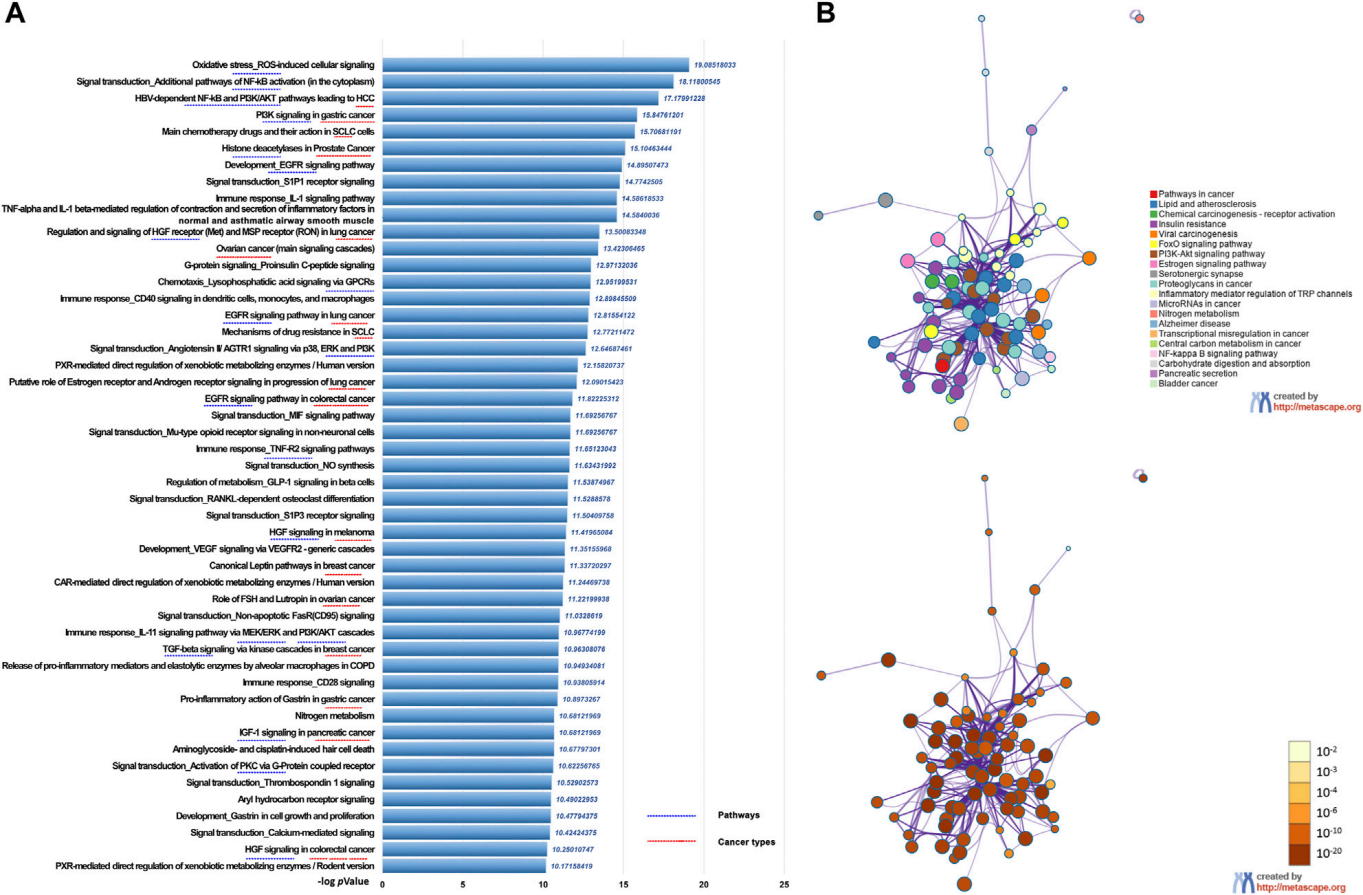
Functional enrichment analysis of CR target genes. **(A)** Functionally enriched pathways of the CR targets genes using Metacore. **(B)** Network of pathways enrichment analysis by Metascape.

We first performed UPLC-MS/MS to identify the characteristic compounds in CR granules. Cinnamaldehyde, 2-hydroxycinnamic acid, coumarin, and 2-methoxycinnamic acid were identified from CR granules (Supplementary Figure S2). To investigate the pharmacological action mechanism of CR, we collected 341 target genes for 83 compounds identified in CR (Supplementary Table S1). Then we inputted these 341 genes into the Metacore database to perform the pathway enrichment analysis. We found that these genes were closely related to various cancer types, including prostate cancer, SCLC, HCC, lung cancer, ovarian cancer, pancreatic cancer, breast cancer, melanoma, and gastric cancer. In addition, they were also involved in many critical cancer-related pathways, including EGFR, TGF-β, and PI3K/AKT signaling pathways (Figure 2A). We also performed KEGG pathway enrichment analysis in Metascape to confirm the Metacore enrichment results, and Pathways in cancer was the top enriched pathway (Figure 2B). These results suggested that CR might mediate multiple pathways in various cancers, which motivates us to investigate CR’s common effects in different cell types.

### 3.2 Differentially expressed genes analysis across ten cancer cell lines treated with CR

**FIGURE 3:**
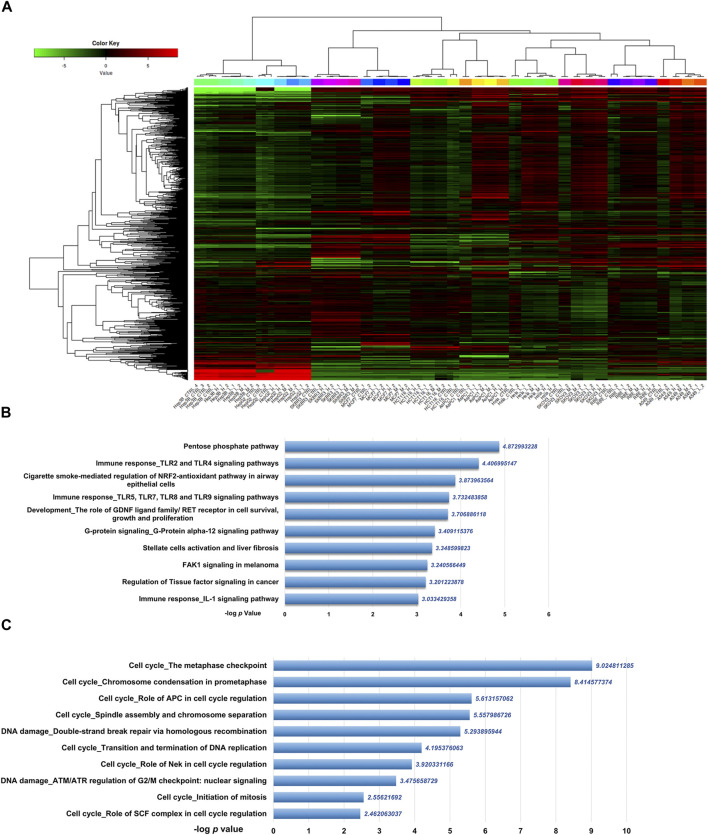
Differentially expressed genes analysis of transcriptomic data from ten cancer cell lines treated with CR. **(A)** Heatmap was used to show the expression profiles of mRNAs. L, Low dosage; M, M dosage; H, High dosage. **(B)** Up and **(C)** down DEGs functional enrichment analysis from ten cancer cell lines treated with three concentrations using Metacore.

To get the mRNA profiles of CR-treated cells, we first chose ten commonly used cell lines in our lab (including A549, AsPC1, HCT116, HeLa, Hep 3B, Hep G2, MCF7, RBE, SKBR3, and SKOV3) and performed CCK8 assay to detect the IC_50_ of the CR in different cell lines. Then, we treated ten cancer cell lines with different concentrations of CR as L (Low dosage, IC_10_), M (Medium dosage, IC_30_) and H (High dosage, IC_50_) group (Supplementary Table S2). After mRNA sequencing, we used iDEP9.3 (http://bioinformatics.sdstate.edu/idep93/) to process the sequencing data, and hierarchical clustering results showed that the heatmap could be clustered into ten different cell lines (Figure 3A). Then we got common DEGs under three concentrations (Supplementary Table S3) for each cell line (Supplementary Figures S3, S4) and further acquired the common DEGs (upregulated, 204 genes; downregulated, 118 genes) (Supplementary Table S4) across 10 cell lines. These final DEGs were inputted into Metacore for functional enrichment analysis. And results showed that upregulated genes were enriched in the Pentose phosphate pathway, and downregulated genes were enriched in the cell cycle process (Figures 3B, C).

### 3.3 Gene set enrichment analysis of the transcriptomic data

**FIGURE 4:**
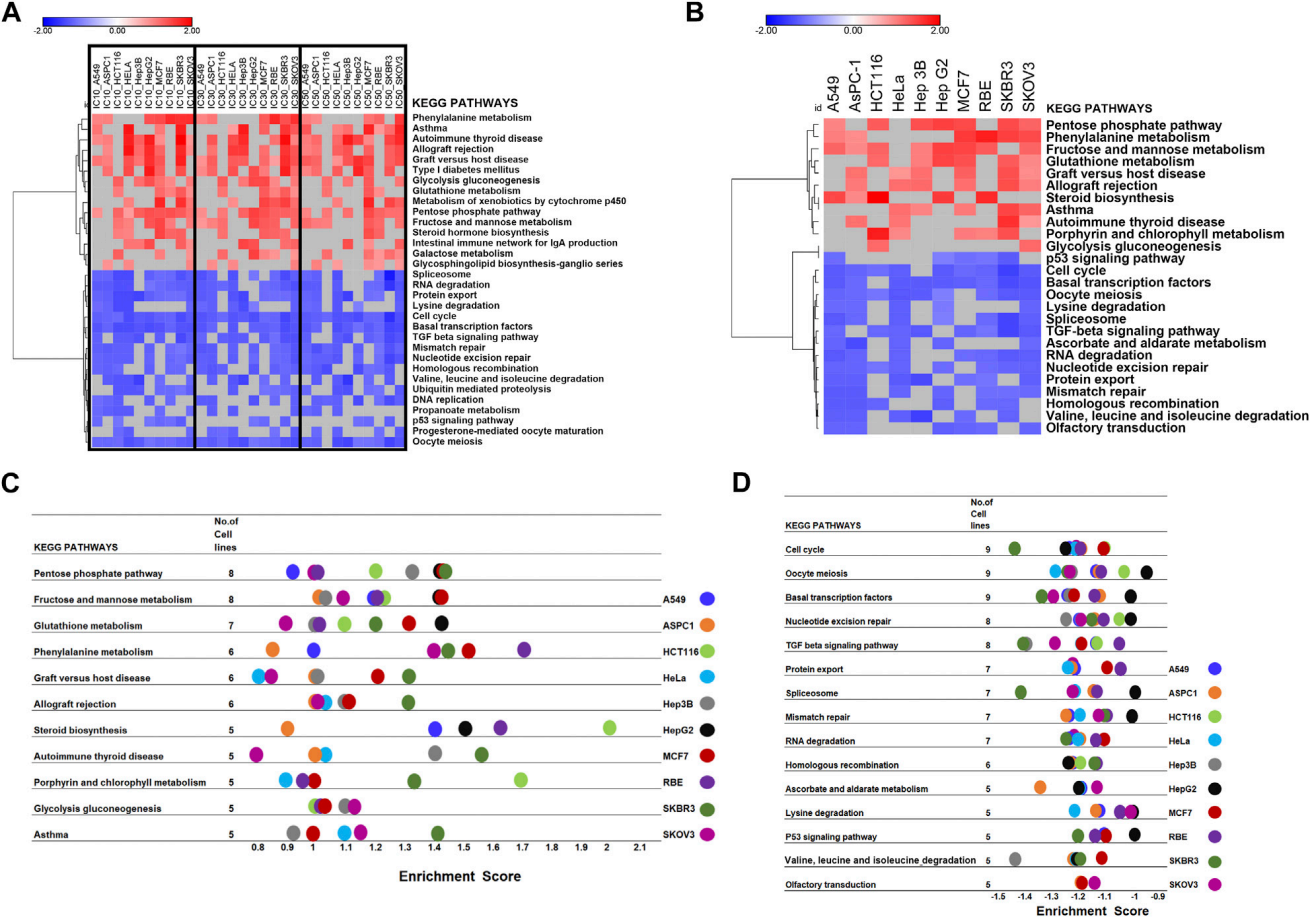
GSEA of the transcriptomic data to screen the critical pathway affected by CR among ten cancer cell lines. **(A)** Hierarchical clustering of enriched pathways from the first GSEA enrichment step was inputting expression profile at the same concentration for 10 cell lines. **(B)** Hierarchical clustering of enriched pathways from the second GSEA enrichment step was inputting different concentration expression profiles for each cell line as three replicates. **(C,D)**. Commonly enriched up- and downregulated pathways from the second GSEA enrichment step, treating expression profiles under different concentrations in each cell line as three replicates to explore the CR effect in different cell lines.

The GSEA tool was further used for the pathway enrichment analysis to confirm DEGs analysis results (Figure 1; Supplementary Figure S1). For the GSEA enrichment analysis, we first inputted the expression profiles at the same concentration for 10 cell lines separately. This step was to verify whether different CR concentration treatments affect different pathways in 10 cell lines. We collected each cell line’s top 20 enriched pathways at each concentration and the corresponding normalized enrichment scores (Supplementary Table S5). Then at each concentration treatment, the most common pathways that appeared in at least 5 cell lines (*n* ≥ 5) were included for further analysis. (Supplementary Tables S6, S7). Then we combined the pathways from three concentrations (Supplementary Table S8). Then we used these pathways combined from three concentrations and their enrichment scores to plot the heatmap to compare the pathway enrichment pattern at different concentrations. The heatmap showed different treatment concentrations across ten cancer cell lines presenting similar pathway enrichment patterns (Figure 4A).

Next, we treated the different concentrations as three replicates based on the first step since their pathway enrichment patterns were similar. Then we moved to the second step. For each cell line, we inputted expression profiles at different concentrations as three replicates into GSEA to find the common influenced pathway across ten cancer cell lines. Also, we collected the top 20 enriched pathways and their enrichment scores for each cell line (Figure 4B). We found that the upregulated pathways influenced by CR across ten cancer cell lines included Pentose phosphate pathway and Fructose and mannose metabolism. In comparison, downregulated pathways affected by CR across ten cancer cell lines included the Cell cycle, Oocyte meiosis, and Basal transcription factors (Figures 4C, D; Supplementary Figure S5). Besides, for RBE cells, upregulated pathways by CR included Phenylalanine metabolism and steroid biosynthesis pathways. For HCT116 cells, upregulated pathways by CR included Steroid biosynthesis and Porphyrin and chlorophyll metabolism. For SKBR3 cells, downregulated pathways by CR included Cell cycle, TGF beta signaling pathway, and Spliceosome (Figures 4C, D).

### 3.4 Identification of hub genes related to cell cycle pathway

**FIGURE 5:**
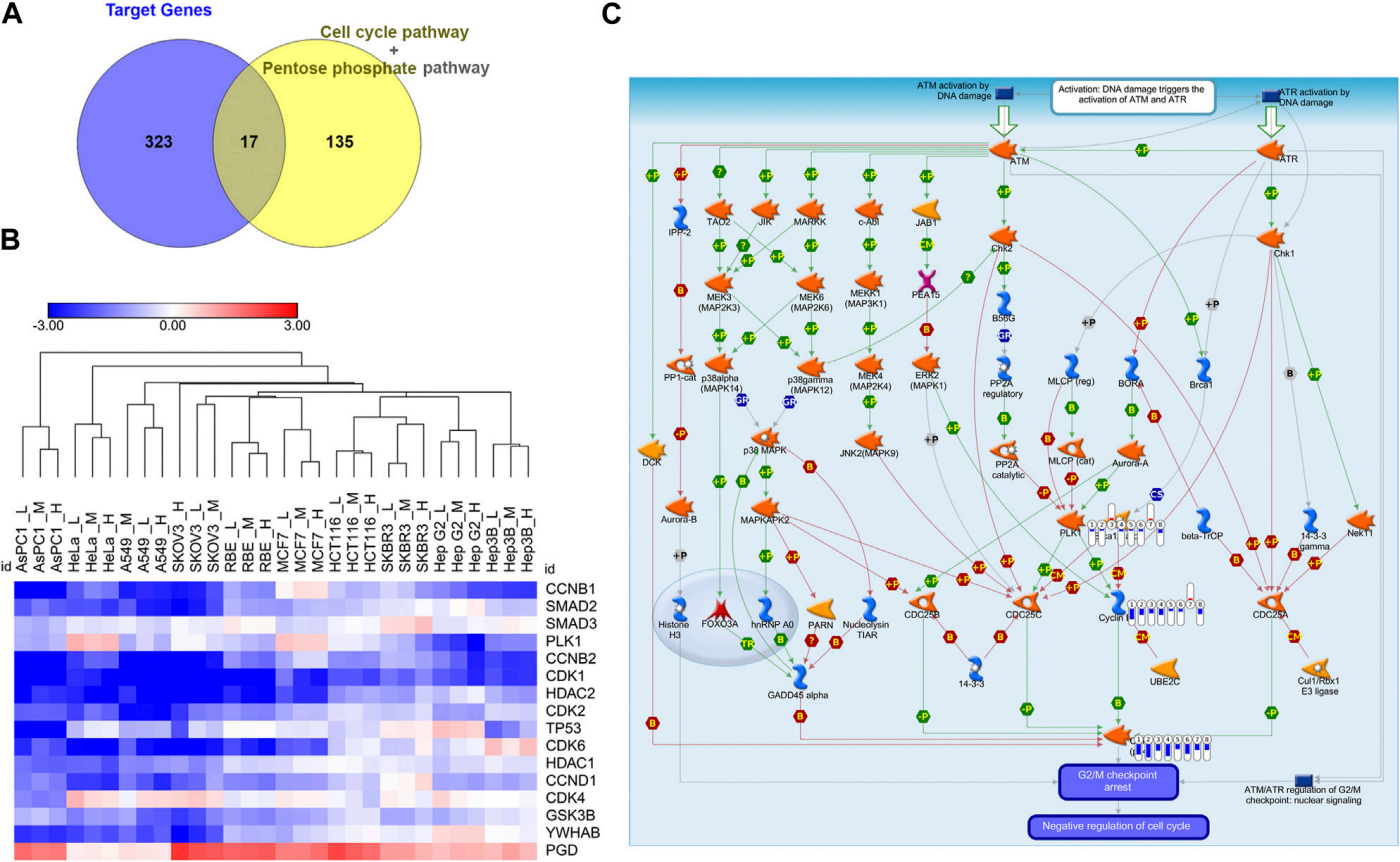
Identification of G2/M cell cycle arrest as the hub targeted pathway by CR. **(A)** The intersection of the Cell cycle and Pentose phosphate pathways-related genes with CR targets genes. **(B)** Log2 fold change heatmap of 16 genes among CR-treated cancer cell lines. Blue, downregulated; Red, upregulated. **(C)** PLK1, CDK1, and CCNB enriched in the DNA damage_ATM/ATR regulation of G2/M checkpoint using Metacore (*p* = 1.056*10^−6^). Red and upward thermometers indicate upregulated genes in the corresponding cell line; Blue and downward thermometers indicate downregulated genes in the corresponding cell line. The thermometer level was the mean log2 fold changes of CR treatment groups in each cell line.

Since Cell cycle and Pentose phosphate pathways have been identified as core pathways affected by CR in ten cell lines with DE analysis and GSEA, we would like to further find the hub genes in these two pathways affected by CR. We obtained the Cell cycle and Pentose phosphate pathway-related genes from GSEA and intersected them with CR targets genes. There were 17 CR target genes overlapped with genes associated with Cell cycle and Pentose phosphate pathway. One gene (PGD) from Pentose phosphate pathway and 16 genes from Cell cycle pathway (Figure 5A). We removed CCNB3 due to its low expression levels among ten cancer cell lines, then the log2 fold change (CR treatment VS. CTRL) heatmap of the remaining 16 genes was shown in Figure 5B. These genes were significantly downregulated in most cancer cell lines except for gene PGD. Then we inputted these 16 genes into Metacore and found that PLK1, CDK1, CCNB1, and CCNB2 were enriched in the G2/M checkpoint pathway (Figure 5C). These results suggested that CR might induce the G2/M checkpoint arrest by suppressing the PLK1/CDK1/Cyclin B axis in cancer cell lines.

### 3.5 Clinical significance and prognosis value of hub genes

**FIGURE 6:**
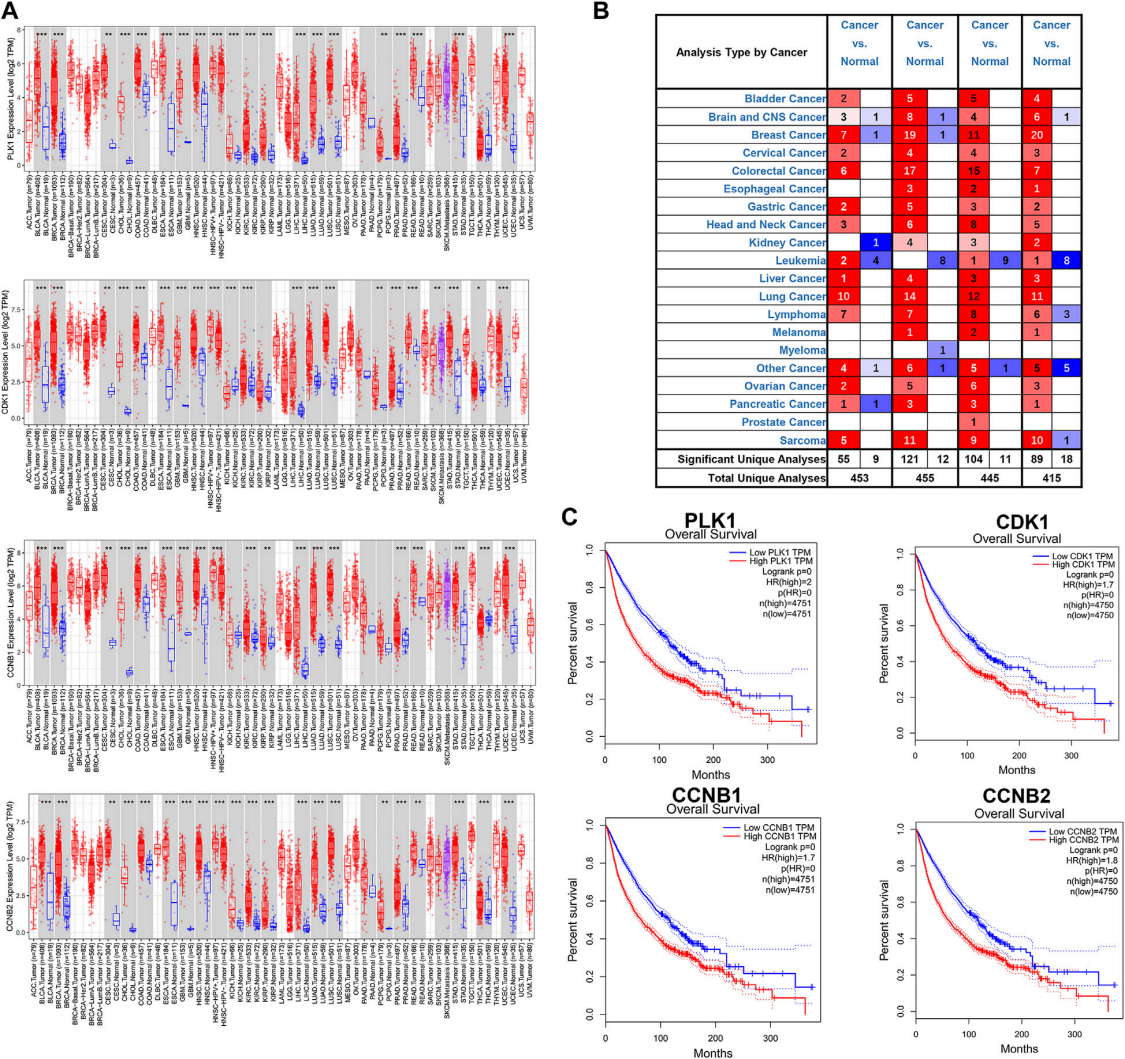
Expressions of hub genes in different cancer types from TCGA and Oncomine **(A)** mRNA expression levels of PLK1, CDK1, CCNB1, and CCNB2 across various cancer types of TCGA dataset using Timer 2.0. The red box was the cancer sample, whereas the blue box was the normal sample. **(B)** mRNA expression levels of PLK1, CDK1, CCNB1, and CCNB2 in different cancer types from the Oncomine database. The number in each unit was the number of data sets. Upregulation was marked in red, and downregulation was blue. *p*-value, 10^−4^; fold change, two; gene ranking 10%. **(C)** The association of PLK1, CDK1, CCNB1, and CCNB2 with overall survival in all cancer types in GEPIA. TPM, Transcripts per Million. The plots were achieved using the GEPIA web server. Data are presented as the hazard ratio with a 95% confidence interval.

**FIGURE 7:**
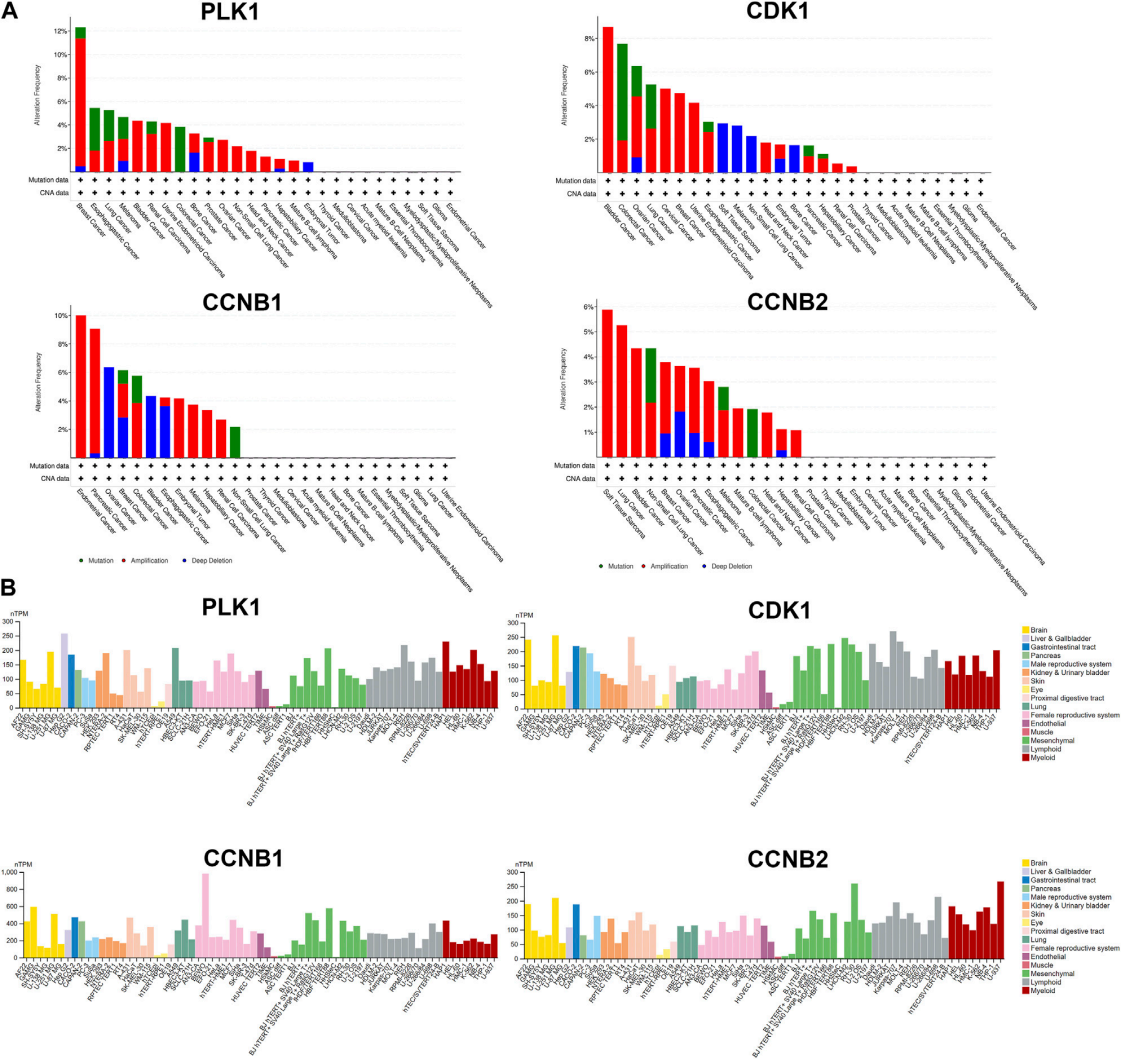
Genetic aberration and cell lines specificity of hub genes. **(A)** Genetic alteration analysis of hub genes using cBioPortal. Mutation, amplification, and deep deletion events were identified in a pan-cancer analysis of whole genomes (ICGC/TCGA, nature 2020), which includes 2,565 patients/2,683 samples. **(B)** RNA expression data as normalized transcript per million (nTPM) values of tissue culture cell lines from human protein atlas (HPA).

Several databases and web servers were used to evaluate hub genes’ clinical significance and prognosis value, including Timer 2.0, GEPIA, Oncomine, HPA, and cBioPortal. Using Timer 2.0, we found that PLK1, CDK1, CCNB1, and CCNB2 were upregulated in different cancer types compared with normal samples from the TCGA data set (Figure 6A). And they were also upregulated in tumors in the Oncomine dataset (Figure 6B). Besides, the low expression levels of these four genes showed a better overall survival rate in different cancer types from TCGA using GEPIA (Figure 6C). We also performed the pair-wise gene expression correlation analysis of these four genes; their gene expression correlations were above 0.8 (Supplementary Figure S6). Then we used cBioPortal to analyze the genetic aberration of four hub genes in tumors (Figure 7A). We chose the TCGA pan-cancer panel, which includes 2,565 patients/2,683 samples (Consortium, 2020). Amplification was the most common genetic alteration in various cancer types, which might be related to their higher expressions in cancer samples. Then we wanted to clarify the cell specificity of the hub genes in different cancer cell lines. In HPA, we checked the RNA expression data of PLK1, CDK1, CCNB1, and CCNB2 values (nTPM) of different tissue culture cell lines. The analyzed cell lines are divided into 16 color-coded groups according to the obtained organs. The four hub genes showed low specificity in various cell lines (Figure 7B).

### 3.6 CR inhibited the expression of hub genes in three chosen cancer cell lines

**FIGURE 8:**
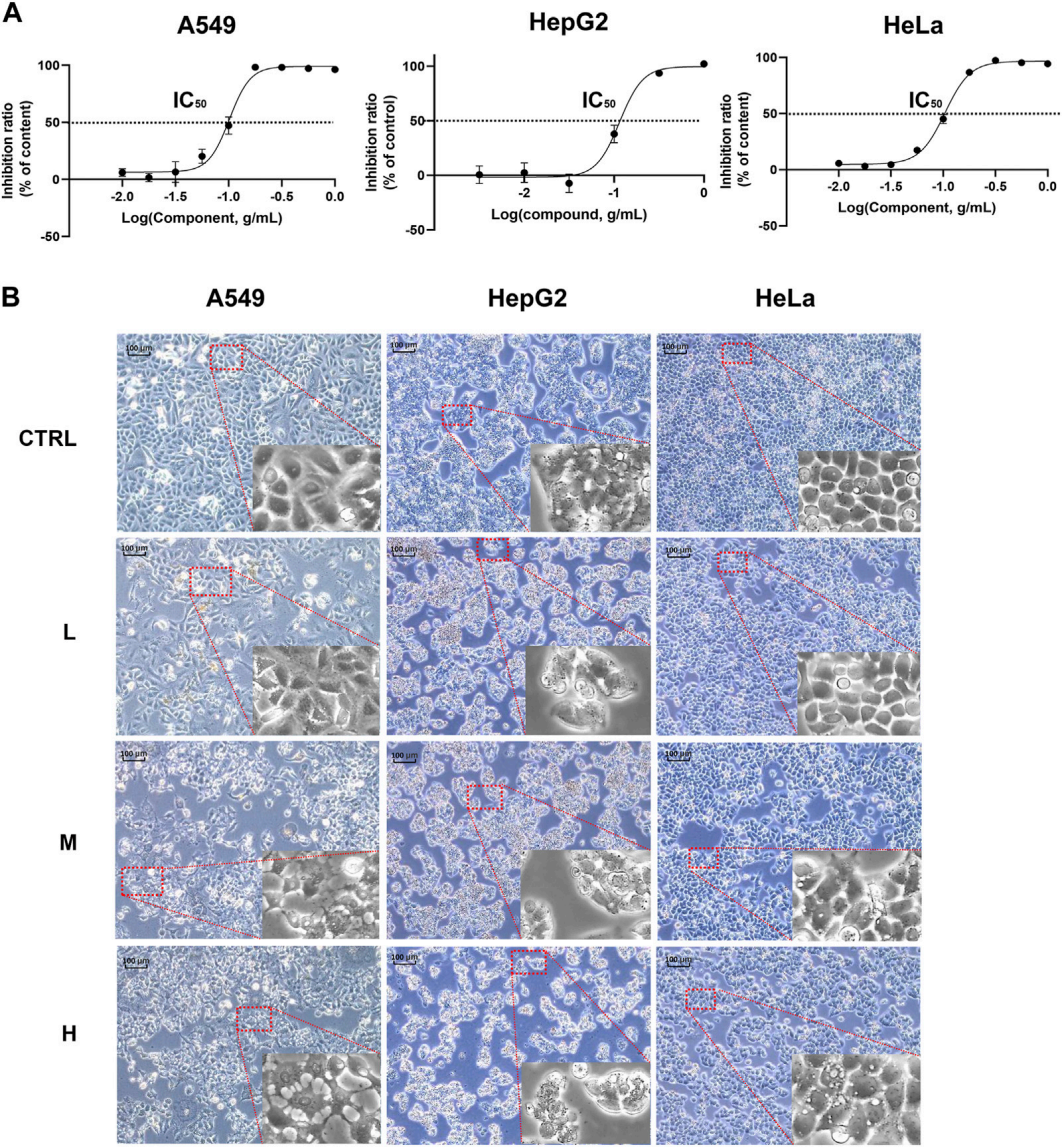
CR inhibited A549, Hep G2, and HeLa cell growth. **(A)** A549, Hep G2, and HeLa cell viability were determined with CCK8 assay. **(B)** A549, Hep G2, and HeLa morphology changes after CR treatment with 24 h.

**FIGURE 9:**
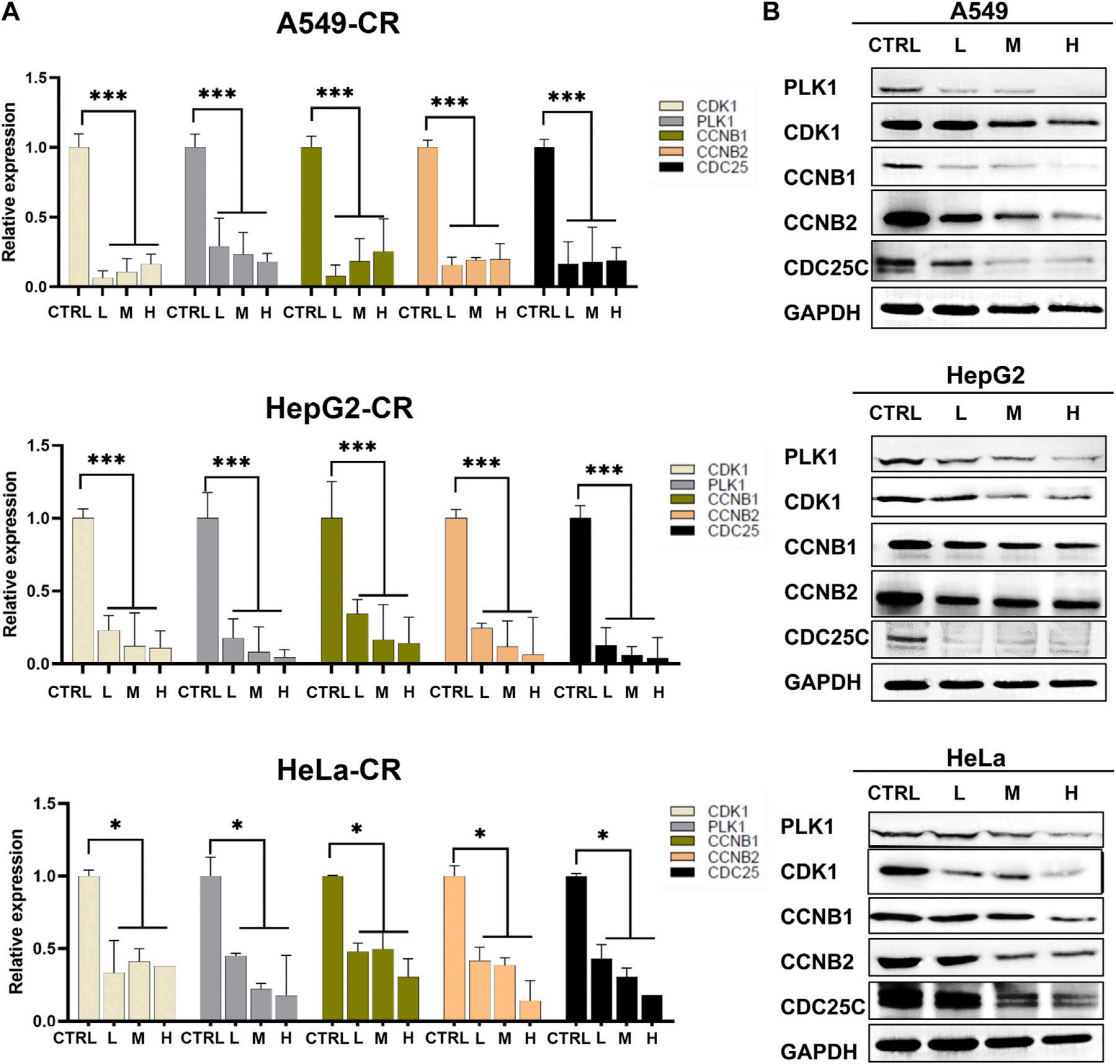
Hub genes expression in A549, Hep G2, and HeLa cell lines treated by CR. **(A)** mRNA expression changes of hub genes after CR treatment. **(B)** Protein expression changes of hub genes after CR treatment. *, *p* < 0.05 vs. CTRL; **, *p* < 0.01 vs. CTRL; ***, *p* < 0.001 vs. CTRL.

Since these genes are low cell line specificity, we randomly chose A549, HeLa, and Hep G2 cell lines for the experimental validation. After 24 h CR treatment, the H group showed many floating dead cells and decreased cell adhesion (Figures 8A, B). From Figure 5C, CDC25C is an essential link in the PLK1/CDK1/Cyclin B axis, and its expression was downregulated across ten cancer cells. Therefore, we added CDC25C in the following *in vitro* experiments. qPCR results showed that all the hub genes were significantly downregulated (Figure 9A). Western blot results also demonstrated that protein expression levels of hub genes were downregulated (Figure 9B). In addition, we also found that PLK1, CDK1, CCNB1, and CCNB2 were upregulated in lung cancer, liver cancer, and cervix cancer tissues from HPA (Supplementary Figure S7). These results indicated that CR could significantly inhibit cancer cell proliferation by inhibiting G2/M checkpoint proteins PLK1, CDK1, CCNB1, CCNB2, and CDC25C.

### 3.7 CR induced the G2/M phase arrest

**FIGURE 10:**
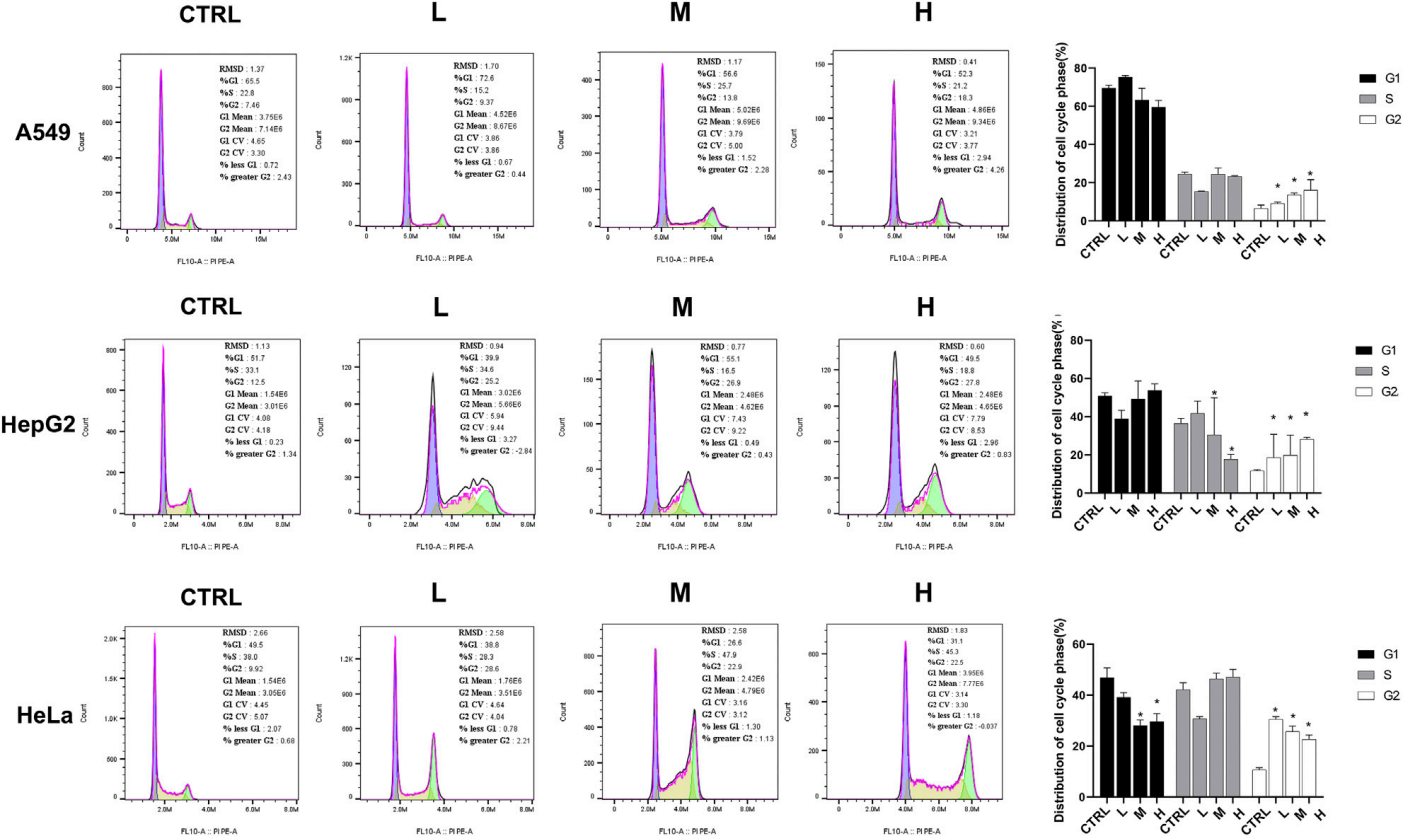
CR increased cancer cells at the G2/M phase and impeded the transition from G2 to M phase. A549, Hep G2, and HeLa cells were treated with CR for 24 h and harvested for PI-staining and flow cytometric analysis. *, *p* < 0.05 vs. CTRL.

The flow cytometry assay was performed 24 h after the CR treatment in A549, HeLa, and Hep G2 cell lines to validate the cell cycle arrest by CR. As shown in Figure 10, the G2/M population of CR-treated cells was significantly increased. For A549 cells, the cell ratios at the G2/M phase were 6.51% ± 1.81% (CTRL), 9.18% ± 0.65% (L group), 13.63% ± 1.03% (M group), and 16.07% ± 5.47% (H group), respectively. Meanwhile, the proportion of G0/G1 cells was decreased. For Hep G2 cells, the cell ratios at the G2/M phase were 11.70% ± 0.43% (CTRL), 18.56% ± 9.11% (L group), 19.85% ± 10.54% (M group), and 28.26 ± 0.94 (H group), respectively. Meanwhile, the proportion of S-phase cells was decreased. For HeLa cells, the cell ratios at the G2/M phase were 10.69% ± 0.81% (CTRL), 30.51% ± 1.03% (L group), 25.71% ± 2.13% (M group), and 22.62 ± 1.72 (H group), respectively. Meanwhile, the proportion of G0/G1 cells was decreased. These results further demonstrated that CR arrested the cell cycle in the G2/M phase.

## 4 Discussion

In recent years, TCM has attracted global attention for its promise in treating diseases, especially complex diseases like diabetes, cardiovascular disease, and cancers (Xu et al., 2019b). Many Chinese herbs’ bioactive components show powerful cancer growth-suppressing effects. TCM can work as a complementary therapy for cancer because 1) it can protect normal cells or tissues against the damage caused by chemo/radiotherapy; 2) it works in synergy with chemo/radiotherapy; 3) it reduces inflammation of the surrounding cancerous tissues; 4) improve immunity and body resistance; 5) extend the life and improve the life quality of the patients with advanced cancer (Hsiao and Liu, 2010). In this study, through the gene targets analysis of CR, we discovered that CR is closely related to different cancer types and cancer-related pathways. Then ten cancer cell lines were selected to study the most critical pathway disturbed by CR. We first performed the CCK8 assay to detect the IC_50_ of CR and then used IC_10_ (L), IC_30_ (M), and IC_50_ (H) to treat the chosen cell lines. From our results, ten cancer cell lines showed decreased cell viability. After CR treatment, Hep G2, A549, and HeLa showed significant cell shrinkage and less adhesion.

Differential expression analysis and gene set enrichment are the two most classic analysis methods for transcriptomic data (Alhamdoosh et al., 2017). Differentially expressed genes (DEGs) are genes that undergo mutations or structural changes at the mRNA level under the influence of different environmental conditions and are essential genes that lead to changes in cell status and biological processes. Focusing on DEGs to find biological clues often has limitations (Rahmatallah et al., 2014). For example, no gene may reach the threshold of statistical significance because the unavoidable noise of sequencing technology may cover differences in biological significance. Furthermore, for a long list of DEGs, the biological meaning behind them is not uniform, and the interpretation of these genes may depend randomly on the biological background of the researcher. Besides, a single gene analysis often ignores the effect of the whole pathway since cell biological reaction is often a collaboration of multiple genes. To address these limitations, Broad Institute developed GSEA. Its principle is based on the known biological pathways of gene sets. It compares whether the gene set appears at either end of the rank gene list to determine whether this gene set is associated with a particular phenotype (Myers et al., 2015). Using two methods in combination can overcome the shortcomings of each approach and help researchers screen the core pathways and genes affected by drugs (Ma et al., 2020). Drug-treated cells were sequenced to elucidate the molecular mechanism by which CR inhibits cell growth, regardless of cell line background. DE and GSEA analyses were performed to search for common perturbed pathways across ten cancer types. In this study, through the enrichment of DEGs, we found that the upregulated genes by CR treatment among ten cancer cell lines were enriched in the Pentose phosphate pathway. In contrast, the downregulated DEGs were enriched in the Cell cycle pathway. Then we also used GSEA further to validate the common influenced pathway after CR treatment. The transcriptome matrix in this study is complex, containing the transcriptomes of ten cell lines under three different concentrations of CR treatment, so we needed to perform the GSEA in two steps (Supplementary Figure S1). For the first step, we want to explore whether different concentrations of CR influence different pathways across ten cell lines. For the second step, we consider different treatment concentrations as three replicates for the functional enrichment analysis. Both analysis methods showed that the upregulated pathway included the Pentose phosphate pathway, and the downregulated pathway included the Cell cycle pathway. Next, we used the CR target genes to intersect with the Cell cycle-related genes and Pentose phosphate pathway-related genes. Finally, we got the 16 genes from the intersection, among which 15 genes were associated with the Cell cycle pathway. Besides, the -log *p* values of Cell cycle-related pathways are much larger than that of the Pentose phosphate pathway (Figures 3B, C). Therefore, we hypothesized that the cell cycle pathway is more affected by CR than the pentose phosphate pathway is affected by CR.

Cancer is inevitably related to abnormal regulation of the cell cycle process. Recently, many TCMs have proven that cell cycle arrest is one of the common mechanisms of the toxicity of TCM on cancer cells (Xu et al., 2011). Huganpian (HGP), a traditional Chinese medicine consisting of six herbs, effectively suppressed liver cancer growth with little toxicity. *In vitro* and *in vivo* experiments demonstrated that HGP induced G0/G1 cell cycle arrest by downregulating CDK4, CDK2, and Cyclin E1 (Gao et al., 2019). Another TCM formula Ze-Qi-Tang can also induce G0/G1 cell cycle arrest in non-small-cell-lung cancer cells, which was associated with the upregulation of the p53 pathway (Xu et al., 2019a). Feiyanning formula (FYN), which has been used for lung cancer treatment for more than 20 years, can also induce G2/M cell cycle arrest in lung cancer cells. Curcumin, a pigment extracted from the rhizomes of Curcuma longa has been reported to arrest cells in G2/M phase in NSCLC (Zhang et al., 2019), breast cancer (Hu et al., 2018), neuroblastoma (Ye et al., 2021), and cervical cancer (Wang et al., 2020). Cell-cycle checkpoint is a series of surveillance mechanisms that ensure DNA replication and chromosome allocation in the cell cycle. When abnormal events occur in cell cycle progression, such as DNA damage or disruption of DNA replication, such regulatory mechanisms are activated, inducing the cell cycle arrest until the cells are repaired (Abraham, 2001). While in cancers, these cell checkpoints are always inactivated because of genetic mutation. Our study focused on 16 target genes that overlapped with the Cell cycle and Pentose phosphate pathway-related genes. Metacore enrichment of these genes further helped us screen out four hub genes (PLK1, CDK1, CCNB1, and CCNB2) which are G2/M regulators. According to the Oncomine and TCGA data, PLK1, CDK1, and CCNB are known to be elevated in different cancer types.

**FIGURE 11:**
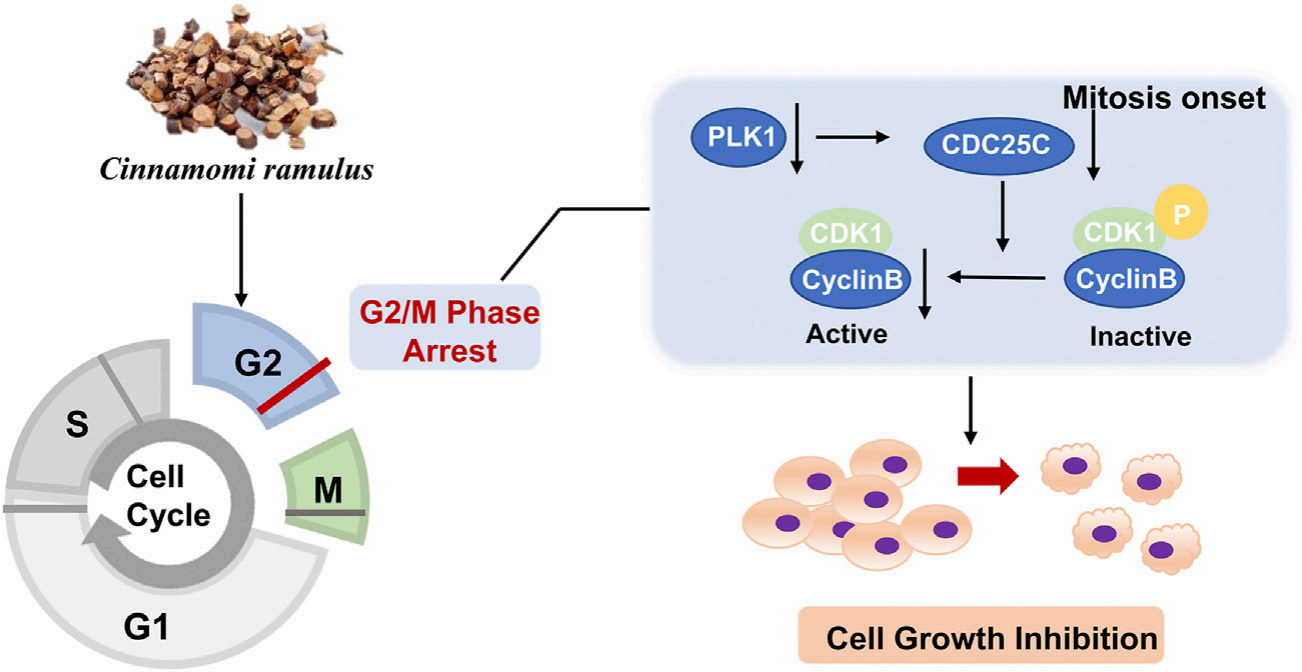
The potential mechanism of CR inhibiting cell growth.

The cell cycle is driven by a complex of CDKs and cyclin (Williams and Stoeber, 2012). Cyclins can bind to a conserved sequence of CDKs molecular structure domains in a non-covalent manner. After CDK activation, it regulates the transition of the cell cycle to different phases (Hochegger et al., 2008). Cyclin B is a cyclin in the G2 phase when cells are preparing to enter mitosis. It is synthesized in the S phase, and the protein is expressed in the G2/M phase. In the G2 phase, Cyclin B combines with the cyclin-dependent kinase CDK1 to form a complex to promote the cell cycle to enter the mitotic phase (Hnit et al., 2020). Cyclin B has two isoforms, Cyclin B1 and Cyclin B2. The expression of its subtypes in tumor cells is significantly abnormal, indicating that it is closely related to the occurrence, diagnosis, and treatment of tumors. PLK1 is a mitotic serine-threonine kinase family member and is an essential kinase for DNA damage checkpoints in G2/M phase (Porter and Donoghue, 2003). PLK1 expression level peaks in G2 and M phases and is highly expressed in cells with active proliferation, like cancer cells. PLK1 phosphorylates CDC25C at multiple sites and promotes its nuclear translocation to increase its activity. After CDC25C is activated, it activates the Cyclin B-CDK1 complex by dephosphorylation and causes the complex translocation into the nucleus to promote the G2/M progression (Liu et al., 2020b). Since CDC25C is the link among CDK1, CCNB, and PLK1, and the expressions of CDC25C among ten cancer cell lines are also downregulated, we added CDC25C for the experiment validation step. Then, because of the low specificity of these genes in different cell lines, we randomly selected 3 cell lines, A549, Hep G2, and HeLa, for the *in vitro* validation experiment. Our study revealed that CR could reduce the expression of the five G2/M phase regulators at both protein and mRNA levels. In addition, the cell cycle assay also demonstrated the increased G2/M cells after CR treatment. CCK8 assay showed that CR could significantly inhibit the proliferation of A549, HepG2, and HeLa cells. Cell cycle results indicated that CR could induce G2/M phase arrest in A549, HepG2, and HeLa cells, and western blot results also confirmed the G2/M phase arrest effects. In addition, Figure 10 showed that cell numbers in the G1 phase decreased in A549 and HeLa with CR treatment, but cell numbers in the S phase did not present significant changes. While in HepG2 cells, G1 phase cells showed no significant change, and the S phase showed a decreased trend with CR treatment. This might be caused by the specificity of different cancer cells and drug incubation time. Therefore, all the analyses and experiments indicated that CR could induce the G2/M arrest by suppressing the PLK1/CDK1/Cyclin B axis, and ultimately inhibiting cancer cell growth (Figure 11).

Cinnamaldehyde is considered to be the characteristic component of CR and has been extensively studied in various diseases. Kim et al. demonstrated that cinnamaldehyde could induce autophagic gastric cell death *via* endoplasmic reticulum stress (Kim, 2022). Cinnamaldehyde has also been proven to inhibit cell growth and promote cell apoptosis in MDA-MB-231 (Liu et al., 2020c). A study on the transcriptome analysis of cinnamaldehyde in non-small cell lung cancer showed that cinnamaldehyde could inhibit cancer cells growth both *in vitro* and *in vivo* (Chen et al., 2020). In our study, UPLC-MS/MS analysis identified cinnamaldehyde from the CR granules. Therefore, we hypothesized that cinnamaldehyde might be the potential active compound which play core inhibitory effects in different cell line. However, this hypothesis needs experiments to validate, which will be included in our future study.

In summary, this study utilized transcriptomic analysis and cellular experiments to investigate the inhibitory effects of CR treatment on ten cancer cell lines. For the first time, we demonstrated that CR treatment could inhibit cancer cell growth by inhibiting the PLK1/CDK1/Cyclin B axis across ten cancer cell lines based on gene expression profile analysis. We used integrated data analysis methods, including DEG analysis, GSEA analysis, and compound target genes analysis, to explore the mechanism of action of CR, which can work as a template for TCM research. In future studies, we will expand the number of cell lines and TCM and use computational modeling techniques (machine learning and deep learning) combined with experiments to clarify the TCM function. Therefore, gene expression profiles in responses to TCM will significantly accelerate the modernization of TCM and provide clues for treating complex diseases.

## Data Availability

The data presented in the study are deposited in the GEO repository, accession number GSE226982 (Available at: https://www.ncbi.nlm.nih.gov/geo/query/acc.cgi?acc=GSE226982).
