# Supplementary figures and images for: Cinnamomi ramulus inhibits cancer cells growth by inducing G2/M arrest
Source: Front Pharmacol. 2023 Mar 17;14:1121799. doi: 10.3389/fphar.2023.1121799 (PMC10063822; doi:10.3389/fphar.2023.1121799)

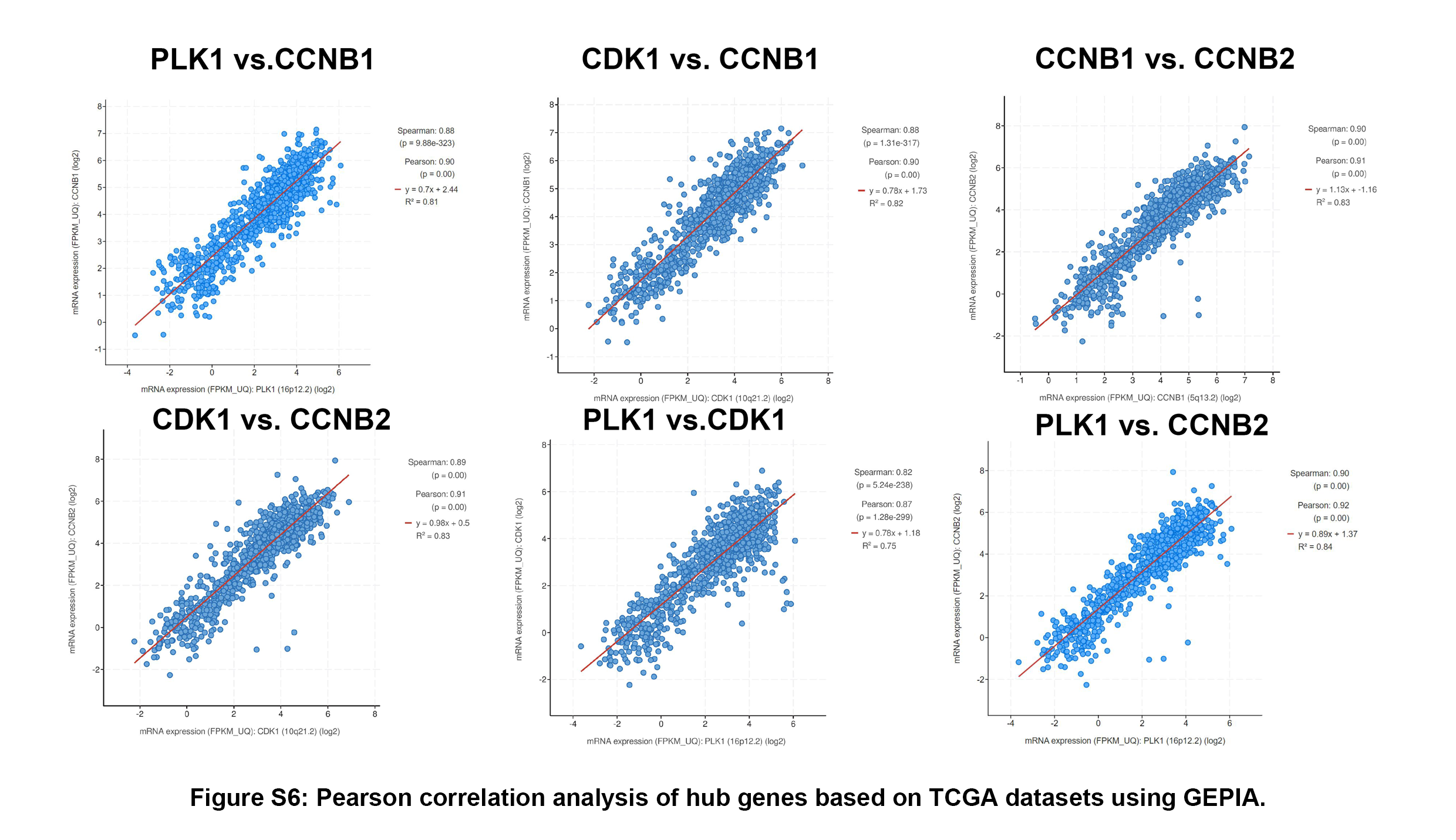

Supplement: Supplementary file 2 [file Image6.tif]

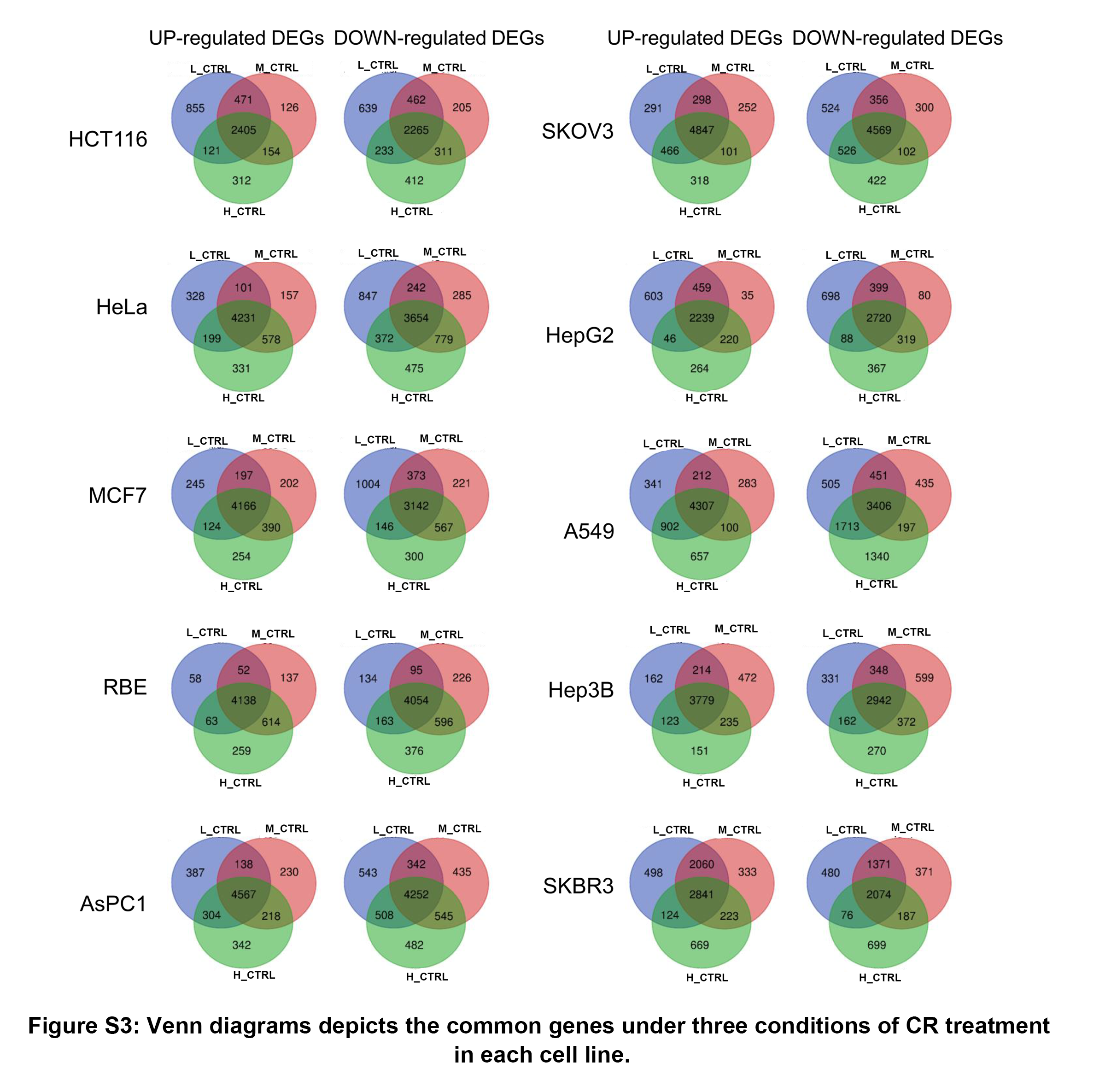

Supplement: Supplementary file 4 [file Image3.tif]

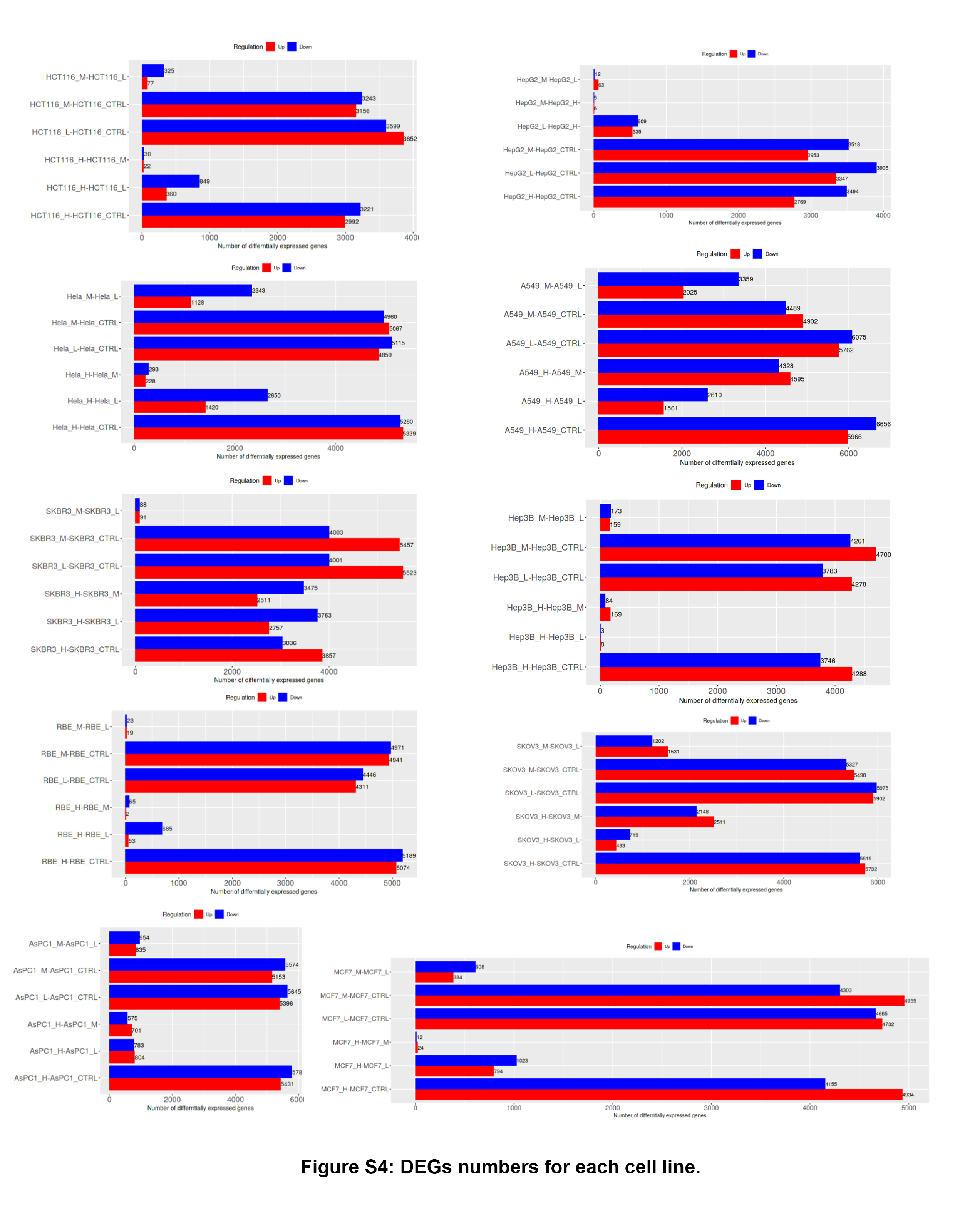

Supplement: Supplementary file 5 [file Image4.tif]

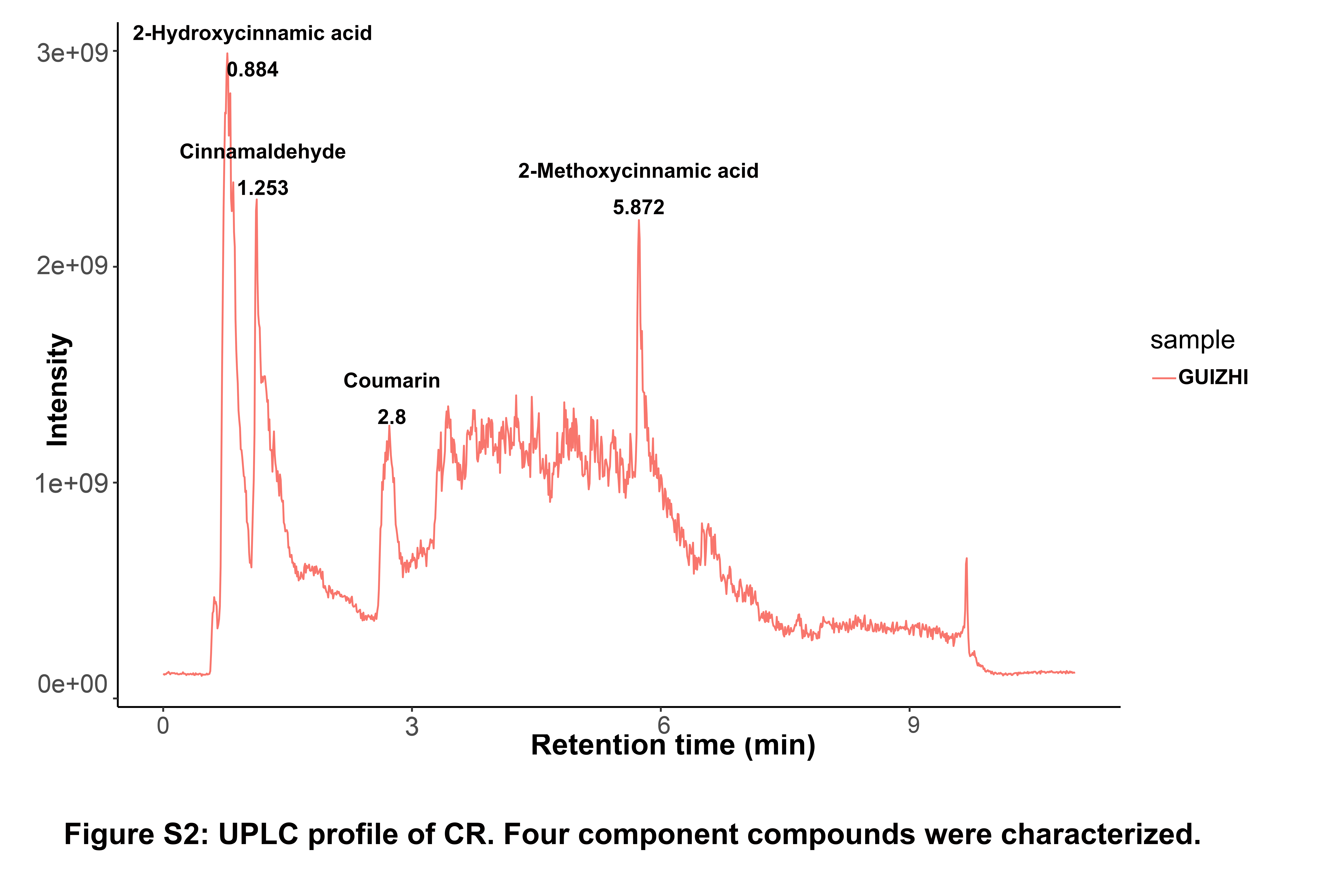

Supplement: Supplementary file 6 [file Image2.tif]

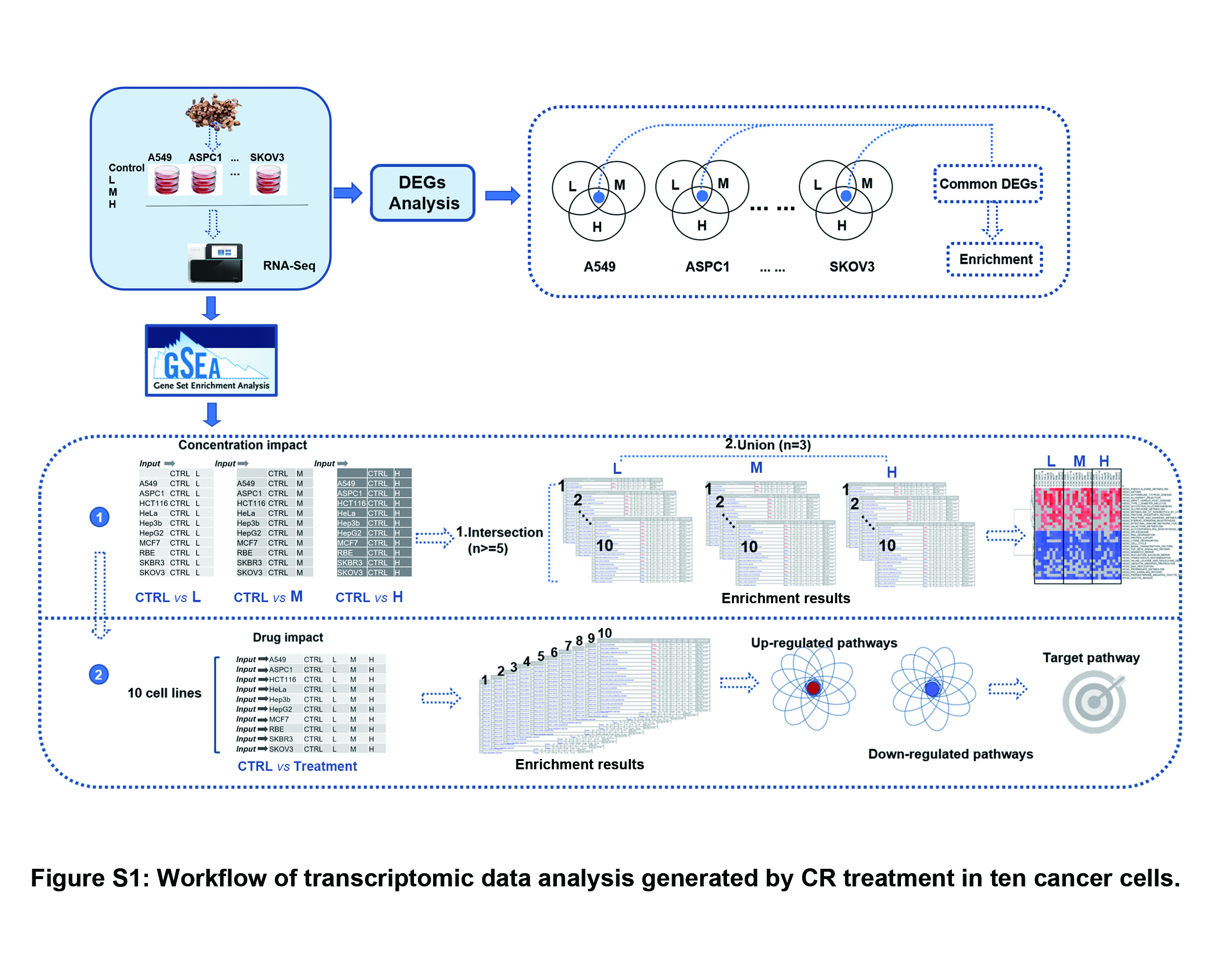

Supplement: Supplementary file 8 [file Image1.tif]

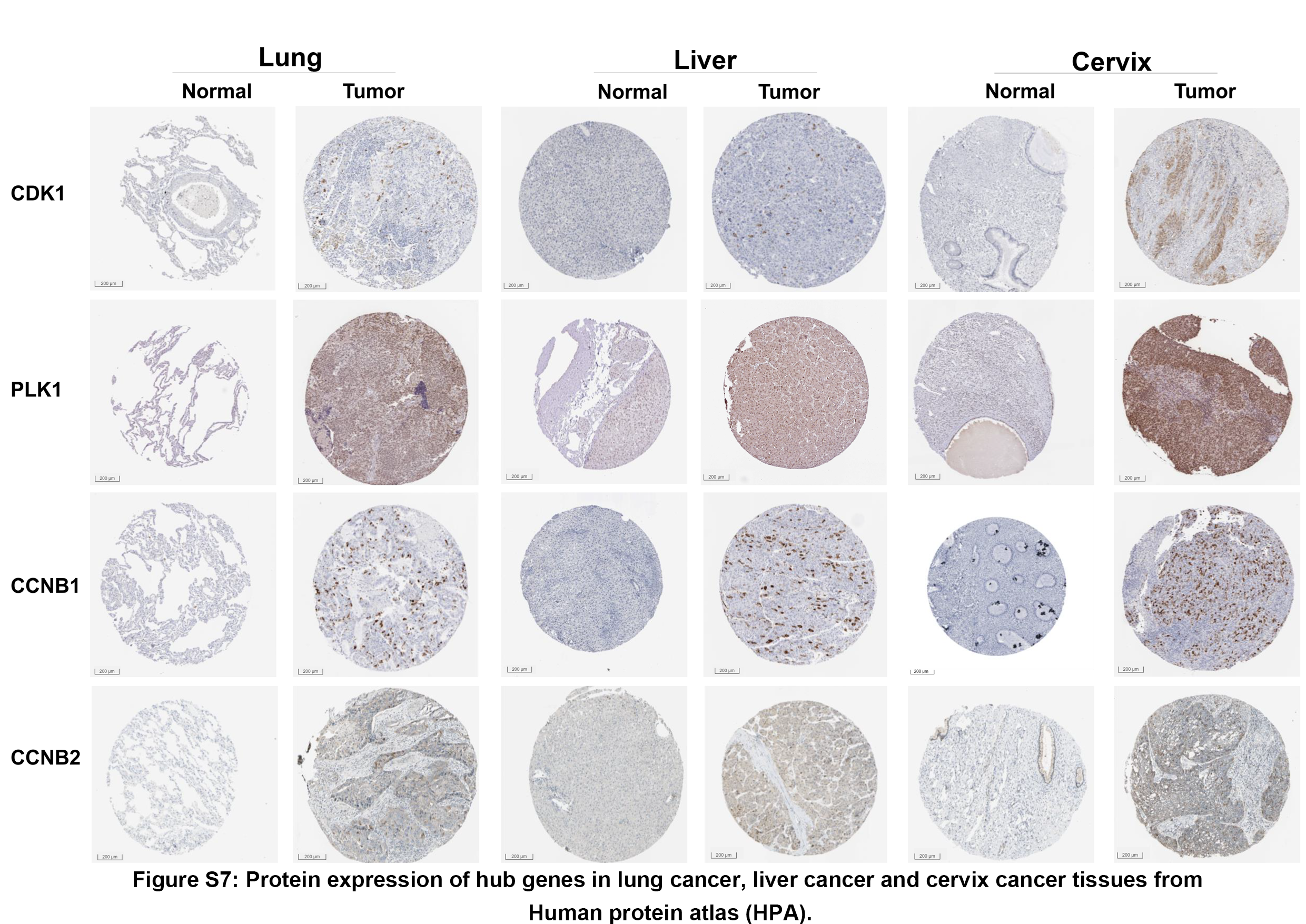

Supplement: Supplementary file 9 [file Image7.tif]

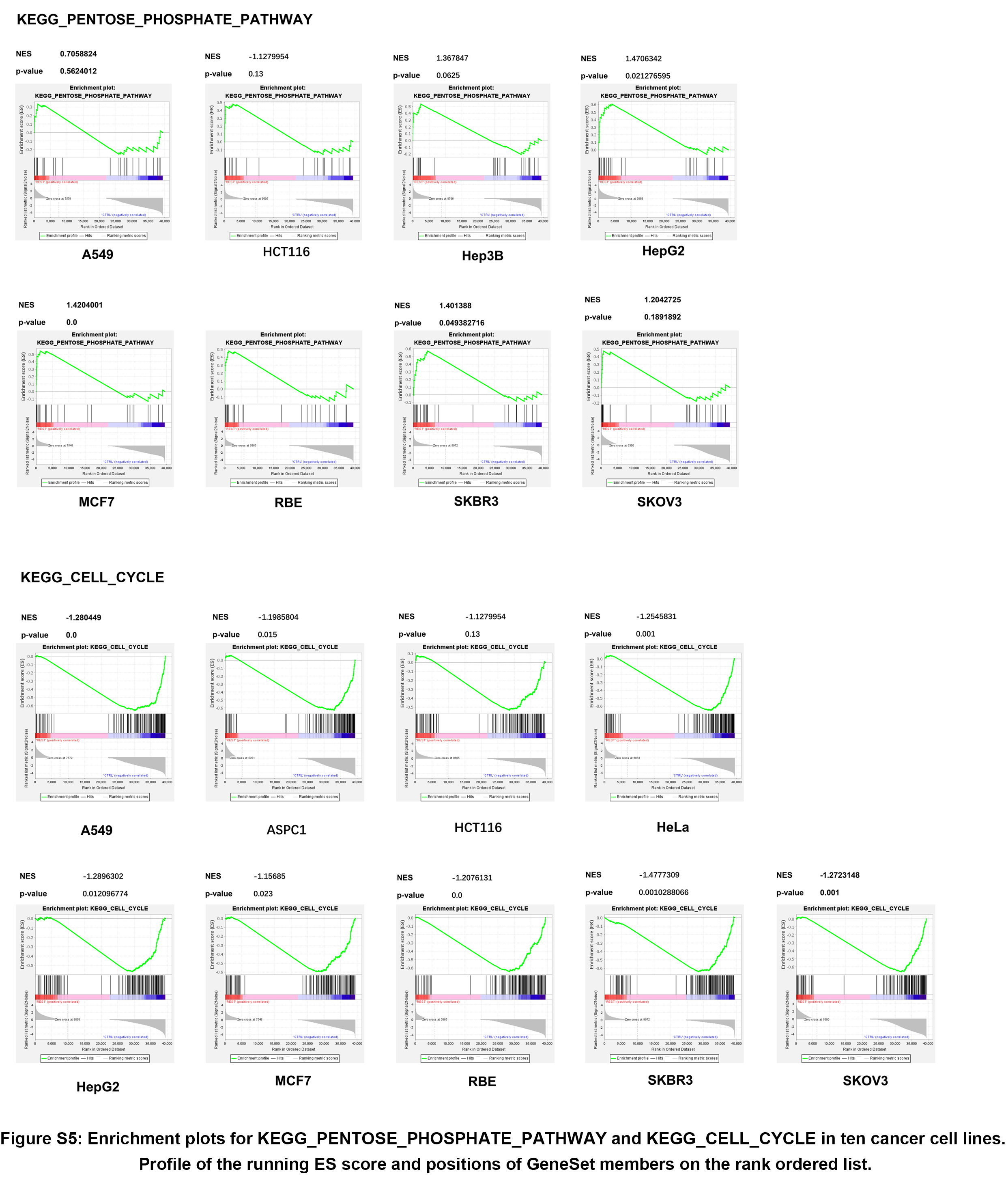

Supplement: Supplementary file 14 [file Image5.tif]
