# Supplementary material for: Cinnamomi ramulus inhibits cancer cells growth by inducing G2/M arrest
Source: Front Pharmacol. 2023 Mar 17;14:1121799. doi: 10.3389/fphar.2023.1121799 (PMC10063822; doi:10.3389/fphar.2023.1121799)
Supplement: Supplementary file 3 [file Table8.docx]

**Table S8**: Combination of pathways disturbed by CR for three concentrations.

| **UP-regulated KEGG Pathways** | **Concentration(s)** |
| --- | --- |
| Fructose and mannose metabolism | L, M, H |
| Allograft rejection | L, M, H |
| Phenylalanine metabolism | L, M, H |
| Graft versus host disease | L, M, H |
| Metabolism of xenobiotics by cytochrome p450 | L, M, H |
| Pentose phosphate pathway | L, M, H |
| Type I diabetes mellitus | L, M, H |
| Autoimmune thyroid disease | L, M, H |
| Glycolysis gluconeogenesis | L, M, |
| Glutathione metabolism | L, M, |
| Galactose metabolism | M, H |
| Asthma | M, H |
| Steroid hormone biosynthesis | L |
| Intestinal immune network for IgA production | L |
| Glycosphingolipid biosynthesis-ganglio series | H |

| **DOWN-regulated KEGG Pathways** | **Concentration(s)** |
| --- | --- |
| Basal transcription factors | L, M, H |
| Spliceosome | L, M, H |
| Valine, leucine and isoleucine degradation | L, M, H |
| Protein export | L, M, H |
| Ubiquitin mediated proteolysis | L, M, H |
| Homologous recombination | L, M, H |
| RNA degradation | L, M, H |
| Oocyte meiosis | L, M, H |
| TGF beta signaling pathway | L, M, H |
| Mismatch repair | L, M, H |
| Cell cycle | L, M, H |
| Nucleotide excision repair | L, M, H |
| Lysine degradation | M, H |
| DNA replication | M, H |
| p53 signaling pathway | L |
| Propanoate metabolism | L |
| Progesterone-mediated oocyte maturation | M |
