# Supplementary material for: Cinnamomi ramulus inhibits cancer cells growth by inducing G2/M arrest
Source: Front Pharmacol. 2023 Mar 17;14:1121799. doi: 10.3389/fphar.2023.1121799 (PMC10063822; doi:10.3389/fphar.2023.1121799)
Supplement: Supplementary file 7 [file Table7.docx]

**Table S7**: Common enriched down-regulated pathways of ten cancer cell lines treated with CR for each concentration (n≥5).

| **No. of cell lines** | **L_DOWN-regulated KEGG Pathways** |
| --- | --- |
| 10 | Oocyte meiosis |
| 10 | Cell cycle |
| 9 | Basal transcription factors |
| 9 | Protein export |
| 8 | RNA degradation |
| 8 | Spliceosome |
| 7 | Mismatch repair |
| 7 | Nucleotide excision repair |
| 7 | TGF beta signaling pathway |
| 6 | Homologous recombination |
| 6 | Ubiquitin mediated proteolysis |
| 5 | Propanoate metabolism |
| 5 | p53 signaling pathway |
| 5 | Valine, leucine and isoleucine degradation |
| **No. of cell lines** | **M_DOWN-regulated KEGG Pathways** |
| 10 | Oocyte meiosis |
| 10 | Cell cycle |
| 8 | RNA degradation |
| 8 | Protein export |
| 8 | Basal transcription factors |
| 8 | TGF beta signaling pathway |
| 7 | Homologous recombination |
| 7 | Spliceosome |
| 7 | Mismatch repair |
| 7 | Nucleotide excision repair |
| 6 | Lysine degradation |
| 5 | DNA replication |
| 5 | Progesterone mediated oocyte maturation |
| 5 | Valine, leucine and isoleucine degradation |
| 5 | Ubiquitin mediated proteolysis |
| **No. of cell lines** | **H_DOWN-regulated KEGG Pathways** |
| 10 | Cell cycle |
| 9 | Basal transcription factors |
| 9 | Oocyte meiosis |
| 8 | Mismatch repair |
| 8 | Nucleotide excision repair |
| 7 | Homologous recombination |
| 7 | Protein export |
| 7 | RNA degradation |
| 7 | TGF beta signaling pathway |
| 7 | DNA replication |
| 6 | Spliceosome |
| 6 | Ubiquitin mediated proteolysis |
| 5 | Lysine degradation |
| 5 | Valine, leucine and isoleucine degradation |
