# Supplementary material for: Cinnamomi ramulus inhibits cancer cells growth by inducing G2/M arrest
Source: Front Pharmacol. 2023 Mar 17;14:1121799. doi: 10.3389/fphar.2023.1121799 (PMC10063822; doi:10.3389/fphar.2023.1121799)
Supplement: Supplementary file 10 [file Table2.docx]

**Table S2**: Drug treatment concentrations detected by CCK8 assay.

| **Cell type** | **CTRL** | **L (mg/ml)** | **M (mg/ml)** | **H (mg/ml)** |
| --- | --- | --- | --- | --- |
| HeLa | 0 | 4.701604 | 6.837098 | 8.648600 |
| Hep G2 | 0 | 5.219251 | 7.578875 | 9.578200 |
| A549 | 0 | 5.171164 | 7.013393 | 8.491667 |
| AsPC1 | 0 | 4.351136 | 6.659417 | 8.366667 |
| MCF7 | 0 | 3.38498 | 5.522501 | 7.61284 |
| SKBR3 | 0 | 3.918974 | 7.165032 | 10.32996 |
| SKOV3 | 0 | 4.036613 | 6.638421 | 9.05236 |
| HCT116 | 0 | 5.140786 | 7.634803 | 11.96219 |
| RBE | 0 | 4.674696 | 6.935288 | 9.11167 |
| Hep3B | 0 | 3.844067 | 6.805907 | 8.98385 |
