# Supplementary material for: Cinnamomi ramulus inhibits cancer cells growth by inducing G2/M arrest
Source: Front Pharmacol. 2023 Mar 17;14:1121799. doi: 10.3389/fphar.2023.1121799 (PMC10063822; doi:10.3389/fphar.2023.1121799)
Supplement: Supplementary file 13 [file Table6.docx]

**Table S6**: Common enriched up-regulated pathways of ten cancer cell lines treated with CR for each concentration (n≥5).

| **No. of cell lines** | **L_UP-regulated KEGG Pathways** |
| --- | --- |
| 9 | Pentose phosphate pathway |
| 8 | Autoimmune thyroid disease |
| 7 | Graft versus host disease |
| 7 | Phenylalanine metabolism |
| 7 | Allograft rejection |
| 6 | Fructose and mannose metabolism |
| 6 | Glycolysis gluconeogenesis |
| 5 | Type I diabetes mellitus |
| 5 | Steroid hormone biosynthesis |
| 5 | Glutathione metabolism |
| 5 | Intestinal immune network for IgA production |
| 5 | Metabolism of xenobiotics by cytochrome p450 |
| **No. of cell lines** | **M_UP-regulated KEGG Pathways** |
| 8 | Graft versus host disease |
| 8 | Pentose phosphate pathway |
| 7 | Fructose and mannose metabolism |
| 7 | Type I diabetes mellitus |
| 6 | Phenylalanine metabolism |
| 6 | Allograft rejection |
| 6 | Autoimmune thyroid disease |
| 6 | Glycolysis gluconeogenesis |
| 5 | Galactose metabolism |
| 5 | Glutathione metabolism |
| 5 | Asthma |
| 5 | Metabolism of xenobiotics by cytochrome p450 |
| **No. of cell lines** | **H_UP-regulated KEGG Pathways** |
| 8 | Graft versus host disease |
| 8 | Autoimmune thyroid disease |
| 7 | Allograft rejection |
| 7 | Pentose phosphate pathway |
| 7 | Asthma |
| 6 | Fructose and mannose metabolism |
| 6 | Type I diabetes mellitus |
| 5 | Phenylalanine metabolism |
| 5 | Glycosphingolipid biosynthesis ganglio series |
| 5 | Galactose metabolism |
| 5 | Metabolism of xenobiotics by cytochrome p450 |
